# Rock carving depth reflects carvers’ geological insight

**DOI:** 10.1371/journal.pone.0358075

**Published:** 2026-09-21

**Authors:** Carina Liebl, Mark Peternell, Christian Horn, Johan Ling, Julian Moyano Di Carlo, Ashely Green, Rich Potter

**Affiliations:** 1 Department of Earth Science, University of Gothenburg, Göteborg, Sweden; 2 Department of Historical Studies, University of Gothenburg, Göteborg, Sweden; Tel Aviv university, ISRAEL

## Abstract

Rock art serves as a remarkable window into Bronze Age societies and is usually interpreted through motif and setting. The exceptionally well-preserved art in Bohuslän is recognized as world heritage with a wide range of imagery engraved into the local Bohus granite. Here we connect the depth of these carvings with near surface structure of this granite by combining ultrasonic pulse-echo B-scans with Structure-from-Motion photogrammetry and DEM based depth modelling for five major panels. The B-scans reveal surface parallel reflective horizons at ~8–9 mm and ~18–20 mm depth (11 and 25 mm at one site) consistent with para-glacial stress-relief microfracture zones. Our investigation shows an average depth for most figures of ~0.96–1.52 mm, and even the deepest carvings lay entirely within the first weak layer. While they exceed the post-glacial depth of weathering (≤1 mm), which was previously thought to be the limiting factor for carving depth. A nearby, polished but uncarved outcrop lacks reflective layers. We conclude that carvers preferentially selected panels with paraglacial weakening and even show preferential placement on an individual panel. Thus, placement and choice of locations were based on geological necessity and its understanding by the carvers.

## Introduction

Nordic Bronze Age rock art is one of the largest collections of rock art in Europe. With the highest density of carvings located in the area of Bohuslän, along the Swedish west coast [1]. Their frequent placement near the sea or other bodies of water is widely interpreted as reflecting the profound maritime orientation of Bronze Age societies [2,3]. The choice of location of individual rock panels has been investigated in relation to their geographical position [3,4] but not by the properties of the rock that was utilized as a canvas. This opens the question of whether the choice of bedrock surface was purely an artistic one or if it was also determined by the properties of the potential canvas.

Some authors have argued that the carvers selected certain rocks for their smoothness and quality [5–7], while others proposed that they were chosen because of their morphological characteristics [2,5,8–11]. Moreover, the mechanical properties of the carved rock—its material response to applied stress—have not yet been considered. Also the depth of figures is a much-overlooked topic which Coles [1] called attention to, warning that the omission of carving depth could affect how the compositions were read. Systematic measurements of depth were taken by authors such as Burenhult [9], Bengtsson [12], and Horn and Potter [13] with the intention of detecting carving techniques and signatures. Where depth is often discussed as an artistic choice and not an affordance of the rock. As an example, Goldhahn [14] analyzed the carvings in Sagaholm, a Bronze Age grave which depicts rock art figures in its sandstone slabs, and maintains that depth differences were used to emphasize certain aspects of the rock art and to drive a narrative. The grave has motifs with different depths, some of which have varying depths between their constructional parts. The deepest section of the motif is usually the central part (the body of animals or humans, the hull of ships, etc.). Since the carvings were made during the burial construction and were then enclosed meaning that they could not have been changed later. Elsewhere it has been proposed that the carvers re-carved certain figures or parts thereof in order to reactivate their symbolic function [15,16]. Either through re-carving processes or by deep initial cuttings, some motifs acquired greater depth and prominence in the compositions [17,18].

Lødøen and Mandt [19] proposed the type of rock and surface weathering as a constraint for the depth of carvings. Zotkina [20] argued that if the rock crust was completely removed, the hardness of the deeper layers could prevent successive carving and there would therefore be a potential maximum depth determined by the characteristics of the rock. The experiments carried out to date have not tested this scenario [21–23].

To address current knowledge gaps concerning the conditions under which rock art was created with regards to west Sweden, this study investigates how the physical properties of the uppermost centimeters of the Bohus granite correlate with carving depth—depths that frequently extend beyond the surface weathering crust. To this end, we used the B-Scan mode of a Pundit PL-200 ultrasonic pulse echo device. In this mode the instrument sends out high-frequency ultrasonic pulses into the granite matrix. These waves are reflected towards an array of multiple receiver by internal features in the rock, e.g., fractures, voids, mineral boundaries, or inclusions. With the reflected waves, the Pundit PL-200 records the depth of these features and renders it into a profile of the granite matrix. The analysis highlights the significance of geological history, particularly the impact of the last glacial period, in shaping the rock surfaces that became suitable canvases for Bronze Age rock art. Furthermore, the study explores how these geological factors influenced the feasibility and practice of carving during the Bronze Age.

### Study area – Bohus granite

**Fig 1:**
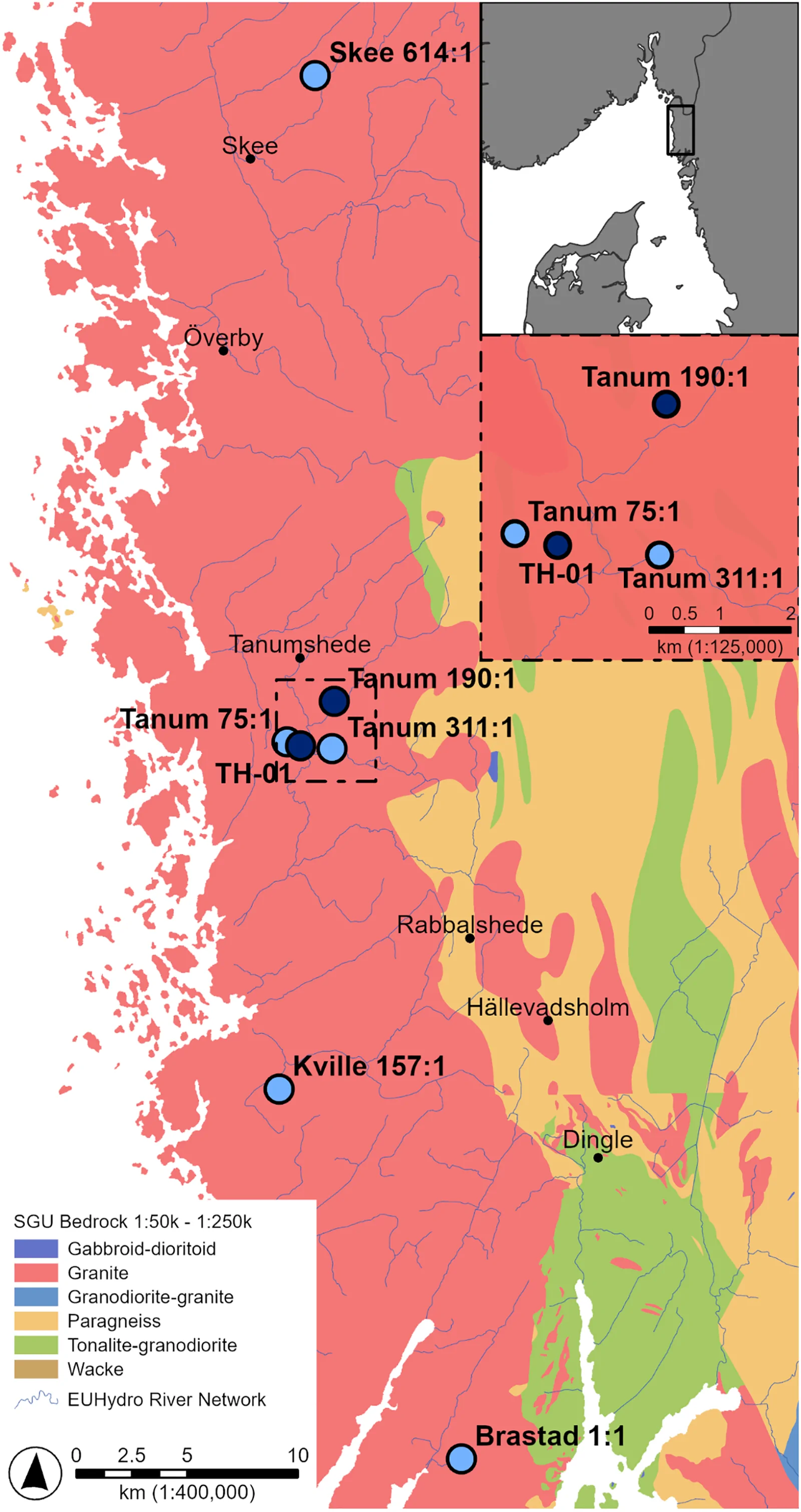
Simplified geological map of the working area. Showing locations of carved rock panels used for this study indicated in light blue. Location of sample sites indicated in dark blue. Basemap data: Berggrund 1:50 000 ©SGU [24]; map top right: Made with Natural Earth.

We focus on the area of Bohuslän due to the exceptionally high density in carvings and their archeological importance. For a good representation of the area 5 locations spread out from north to south (Fig 1) have been chosen, which are fully archeologically documented, have a relevant number (>150) of images (Table 1) and figures with a wide variety, such as depictions of boats, humans, animals, wagons, cupmarks and others. Tanum 75 and Tanum 311 are in the direct area of the world heritage. All sites show carvings made in the regional bedrock called Bohus granite as most of the rock art in the area is.

**Table 1:** Number of data points used for ArcGIS depth calculation and result from statistical analysis. All depth in mm.

Location	Skee 614:1	Tanum 75:1	Tanum 311:1	Kville 157:1	Brastad 1:1
# grid points total	19770	67515	40278	69534	71798
# separated figures	155	369	488	411	323
Single carvings					
Max depth	3.646	3.541	3.662	5.325	7.687
Mean depth	1.101	1.131	0.962	1.516	1.207
Central 50%	0.841-1.517	0.899-1.466	0.561-1.493	1.172-1.938	0.898-1.766
Wisker range	0.314-2.255	0.519-2.284	0.006-2.808	0.672-3.022	0.423-3.046
# outside whisker range	4	4	3	9	16
% deeper than weathering	97.5	93.5	95.8	96.7	100
Overlapping carvings					
Max depth	4.338	5.670	5.571	7.126	7.580
Mean depth	0.942	1.640	0.721	1.484	1.398
Central 50%	0.647-1.913	0.948-2.181	0.312-1.328	0.950-2.139	0.955-2.280
Wisker range	0.024-3.396	−0.037-3.505	−0.109-2.804	−0.002-3.355	−0.395-4.195
# outside whisker range	1	1	4	2	6
% deeper than weathering	83.3	83.3	88.7	79.5	96.1
Cupmarks					
Max depth	7.419	19.770	8.850	7.417	9.040
Mean depth	2.120	1.978	1.317	2.077	1.383
Central 50%	1.486-2.792	1.31-2.854	0.926-2.159	1.457-2.974	0.935-2.155
Whisker range	0.032-4.569	−0.044-4.823	−0.020-3.946	0.373-5.162	−0.049-3.967
# outside whisker range	5	13	11	5	6
% deeper than weathering	98.7	94.0	98.3	95.9	97.7
Weathering depth	0.48	0.70	0.05	0.83	0.07
1^st^ reflective layer	8.22 ± 0.08	8.01 ± 0.06	8.29 ± 0.10	11.03 ± 0.10	8.37 ± 0.07
2^nd^ reflective layer	18.29 ± 0.17	18.23 ± 0.13	18.51 ± 0.21	24.35 ± 0.22	18.67 ± 0.17

Bohus granite spans along the northern part of the coast of western Sweden extending roughly from Lysekil in the south and continuing in the north into Østfold (Norway) where this rock is known as Iddefjord granite. The granite was emplaced post-kinematic at the end of the Sveconorwegian orogeny ca. 925Ma ago following a shallow eastern dipping weakness zone [25–27]. The mineral composition of quartz, k-feldspar and plagioclase plots in the monzogranitic field for the whole area and shows a variable amount of biotite (1%−10%) and in some locations some secondary muscovite. The different magma surges lead to some variation in texture and colors [25]. This composition and the mainly uniform magmatic structure of the granite is a predominant factor for its high mechanical strength. Uniaxial compressive strength (UCS) ranging between 100–158 MPa are reported depending on the amount of mineral defects [28–30]. And a tensile strengths up to 11 MPa [29,31].

### Near surface weathering in the Bohus granite

The present‑day rock surface reflects the combined effects of synglacial, paraglacial and postglacial processes. During the last synglacial, abrasion, plucking and subglacial meltwater removed any earlier near‑surface weathering [32]. Glacial advance and retreat also produced characteristic structures on the surface such as striations, cirques and arêtes. Glacial abrasion, reworking and compaction of fine rock material generated polished surfaces [33,34] that bear an amorphous, silica‑rich coating only a few microns thick. Paraglacial ice retreat in the area approximately 12–12.5 ka ago [32,35] led to surface‑parallel stress release and the formation of near‑surface microfractures without disrupting the thin polished coating [36,37]. Subsequent postglacial physical weathering has produced, for example, flaking/exfoliation, granular disintegration and pitting. Repeated freeze–thaw cycles roughen surfaces and widen existing joints. In parallel, chemical weathering—often enhanced by acidic rainwater—causes surface discoloration that can penetrate to greater depths along joints [38].

Overall, the Bohus granite shows only minimal surface loss due to physical weathering in the range of 0–0.03 mm per 1,000 years, with local spots up to 2.13 mm per 1,000 years associated with granular disintegration and micro exfoliation [38]. Accordingly, synglacially polished surfaces must have undergone minimal to no alteration since the carvings were made. Chemical weathering is represented by discoloration as result of alterations of mica and feldspar minerals and is reported to reach up to 5–10 cm in locations without surface polish [39,40]. No numbers are reported for polished outcrops but are expected to be significantly smaller if glacial polish is remaining. Areas with increased physical and/or chemical weathering are connected to either increased human activity or spruce forests which produce a very acidic litter [38,40].

## Methods

### Field data

#### a) Ultrasonic depth measurements.

Non-destructive ultrasonic soundwave measurements were taken to investigate the depth structure of rocks. At the sites Skee 614:1, Tanum 75:1, Tanum 311:1, Kville 157:1, and Brastad 1:1 (Fig 1) depth profiles of the surface have been measured using the ultrasonic B-scan function of a Pundit PL-200 with a PL-200PE transducer and a frequency of 50kHz [41,42]. The transducer head has a set of 9 transmission and 9 receiver sensors, each arranged in 3x3 squares. The profile is created by sending a soundwave pulse from each of the transmission sensors which is then detected at each receiver sensor. By combining the wave data and arrival time for each sensor an interpolated signal for each point along the profile is automatically determined. The instrument is moved 5 mm along a profile line and the procedure is repeated until a profile length of 9 cm is recorded. For each measurement location a wave velocity estimation was done using the build-in function of the Pundit PL-200. The estimation was done at least 5 times for each location, and the mean velocity was applied for the later depth calculation. Standard deviation of velocity estimated for each location was 2.6% and with repeating each estimation 5 times this leads to a statistical uncertainty of approx. 1.2% (standard deviation/sqrt(#measurements)). For the systematic device error we assume a conservative estimate of 10%, leading to a total uncertainty of 11.2% for depth determination for each step of a measured profile. The error was then propagated to the mean depth for each profile and further to the average depth of each panel.

Depending on the size of the location between 5 (Tanum 311:1, smallest surface area) and 12 (Tanum 75:1, largest surface area) profiles were measured. Individual sampling locations for each panel were chosen randomly around the carved area and, where space allowed it, in-between carvings. An example of distribution of profiles is shown for Kville 157:1 in Fig 3A. Detailed information for placement on each panel, velocities at each location can be seen in S1 File.

#### b) Photogrammetry.

Georeferenced 3D models were created using Structure from Motion (SfM) photogrammetry, a well-established method in the documentation of rock art [43,44]. It is based on the acquisition of sharp photographs -called cameras in this process- of the documented surface with at least 60−70% overlap to allow the algorithm to identify identical points in several cameras. Based on these points and the distance between their location in different cameras the algorithm can reconstruct 3D geometry [45]. Photos were taken with a Canon EOS 90D with a Canon EF-S 18-135/3,5-5,6 IS USM lens, shooting in RAW format and processing in Adobe Camera Raw (v. > 2021). The 3D models were processed in Agisoft Metashape (v. 2.0) using high quality alignment, high quality depth maps, and 4K textures.

### Depth determination

#### a) Ultrasonic wave data.

The wave data from the B-scans was plotted with a MATLAB script and manually inspected to identify whether a reflective surface was present (see S1 File). In case such a surface was identified, times of phase-changes corresponding to arrival of a wave signal calculated with the same MATLAB script were used together with the estimated velocities of each location to calculate the depth of a reflective layer for each step of the profile. The average depth for each location is calculated.

#### b) SfM and ArcGIS.

**Fig 2:**
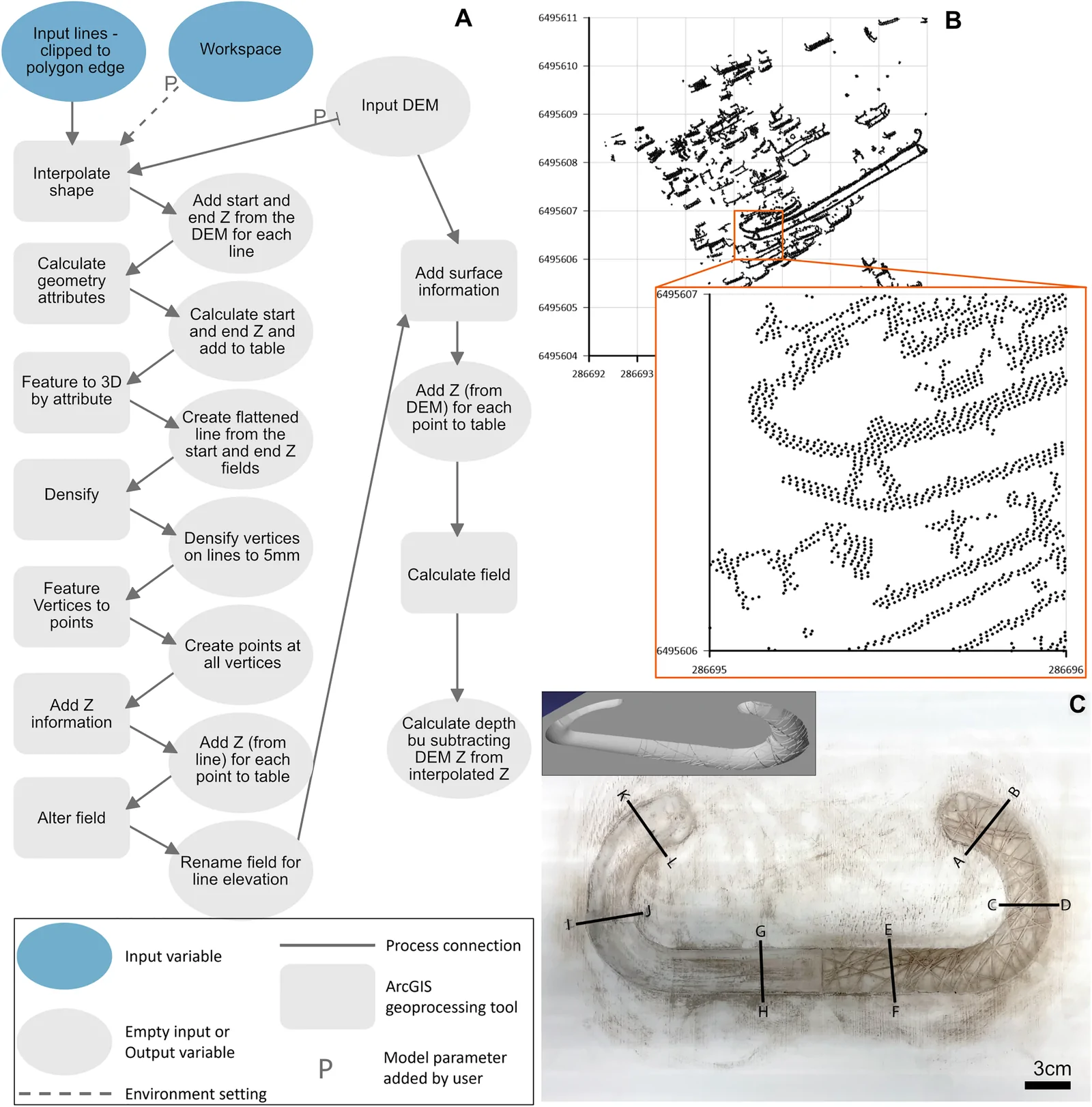
Model for depth calculation and error estimation. A: workflow to calculate the depth of carvings in ArcGIS based on SfM 3D models of the carvings. B: Distribution of grid data points in the motifs as example from Kville 157:1. C: Orthophoto of 3D printed model with known dimensions for error estimation of the calculation procedure. Lines along which error was estimated are indicated. Upper left corner: SfM 3D model of the printed model, made with the same resolution as models from the field.

Using digital elevation models (DEMs) derived from the 3D SfM models, the depth of the carvings was calculated in ArcGIS Pro using the workflow shown in Fig 2A. The input data is the DEM and polygons of the extent of each motif on the panel. The polygons were drawn manually using the DEM and, where necessary, enhanced local relief visualizations as reference. A line was drawn along the slope of the panel and then offset to create a series of lines across the carved surface. These lines were then clipped to the polygon extent. A straight line was drawn using the start and end Z values of the clipped line. This new line is densified with vertices every 5 mm and exported to points. For each point we record the interpolated Z value from the line, estimating the surface elevation before carving, and the current Z value from the DEM. These two values are subtracted to provide an estimated carving depth at each point.

The average of the deepest 10% of data points for each figure were calculated to determine a depth for each figure on the panel. Data assigned to one figure were calculated separately from points assigned to two or more figures (overlapping carvings) and cupmarks.

#### c) Error estimation.

To determine the error range for DEM-based depth calculations the results were verified with two independent methods. Depth measurements were performed in the field on 17 carvings from 7 panels within the study area by using a caliper and then compared the result to the depth measurements in ArcGIS from the same motifs. In addition, a simplified “carving” with known dimensions was 3D printed (Fig 4B) with submillimeter accuracy in shape and depth. A digital 3D model of the printed carving was then prepared using SfM (Fig 4C) with the same resolution as the field models. The depth of the printed model was calculated using the same workflow in ArcGIS and compared to the 3D printed carving geometry. Depth of the printed model was measured at six points extracted from the midpoints of lines drawn on the print that were also visible in the orhthophoto generated from the SfM model (Fig 4C) The results from both verification methods lead to a total calculation error range of ±0.25 mm for the ArcGIS based method.

## Results

### a) Field samples and weathering depth estimates

**Fig 3:**
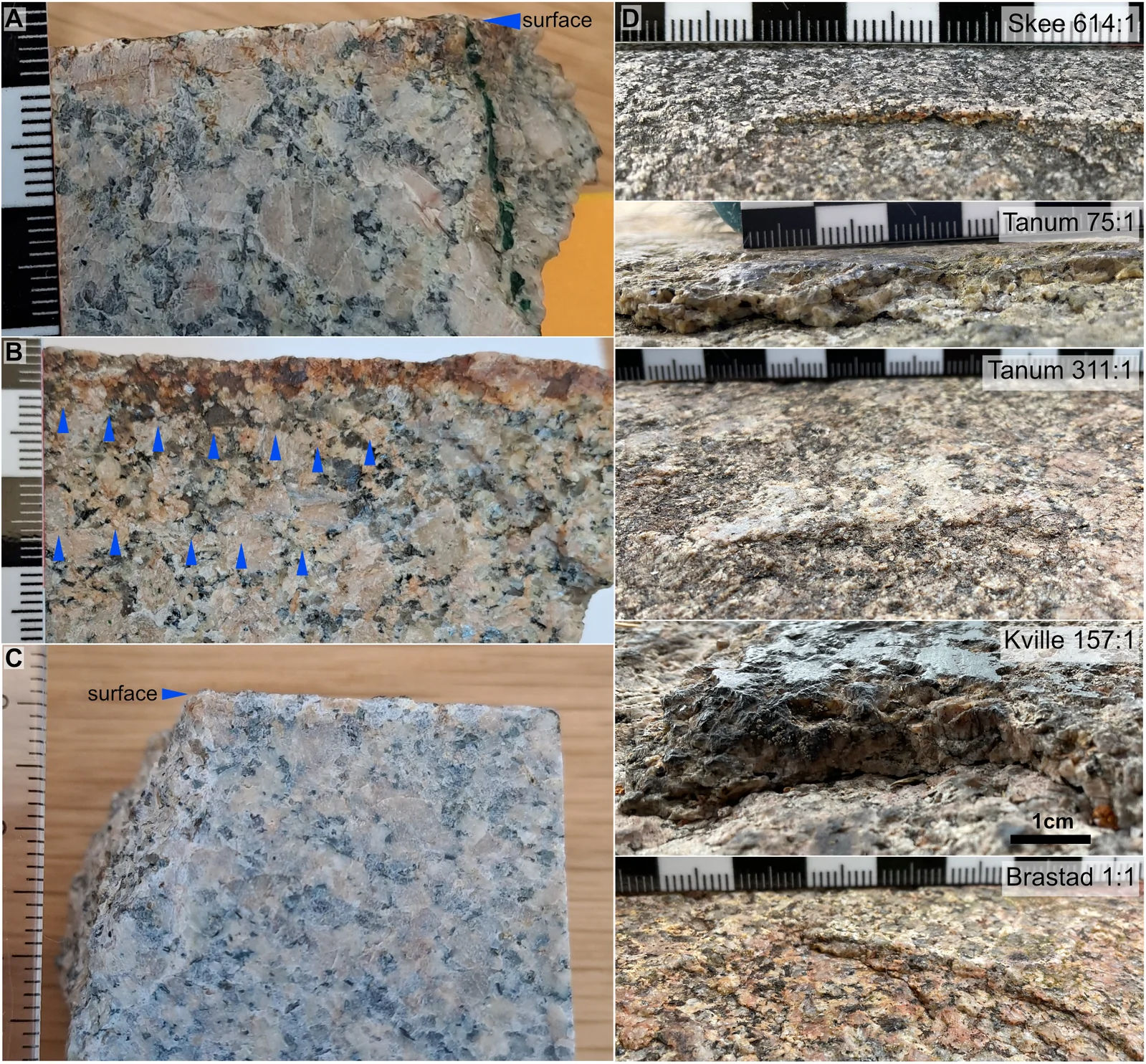
Overview of field samples and surface of the investigated sites. A: granular Bohus granite from Tanum 190:1, fresh cut with weathered/discolored layer. Polished surface indicated with blue arrow. B: granular Bohus granite from Tanum 190:1 (with weathered/discolored layer), cut along a preexisting fracture. Blue arrows indicate depth of discontinuities. C: Sample from location TH-01, outcrop close to Tanum 75:1 showing no discontinuities and little weathering depth, location has no carvings. D: Examples from naturally broken off edges on the investigated panels from which the weathering depth was estimated. Estimates are reflecting maximum weathering depth.

A granular Bohus granite sample taken from Tanum 190:1 (Fig 3A) – a panel with carvings – shows discoloration from postglacial chemical weathering. The weathered layer is approximately 1–5 mm (Fig 3A) and becomes deeper along fractures connected to the surface. Additional discontinuities are found at a depth of 8–9 mm and 19–20 mm (Fig 3B; Table 1), consistent with the depth of a reflective layer identified in an ultrasonic B-scan [46] performed at the site (see methods section). Note that the face seen in Fig 3b follows a pre-existing fracture and has therefore been exposed to weathering, which makes the discolored surface layer appear thicker.

The sample in Fig 3C was taken from an outcrop beside a road close to Tanum 75:1 (location TH-01; Fig 1). This outcrop exhibits an excellent glacially polished surface but was never used for carvings. The discolored layer is approximately 1 mm thick, and no internal discontinuities were observed. B-scans acquired at this location show no reflective layers (S1 File).

Weathering depth estimated from naturally broken edges for TH-01 and Tanum 190:1 is in accordance with respective depth determined from thin sections. Along natural edges progression is expected to be faster and our weathering depth estimated thus reflect maximum depth with an error of ± 0.1 mm. Across all investigated sites weathering depth are estimated from natural broken edges and fall within the expected range, from < 0.1 mm at Tanum 311:1 to 0.83 mm at Kville 157:1 (Table 1). This variability accords with the expected depth of weathering for the regional climate and the time elapsed since the last glaciation.

**Fig 4:**
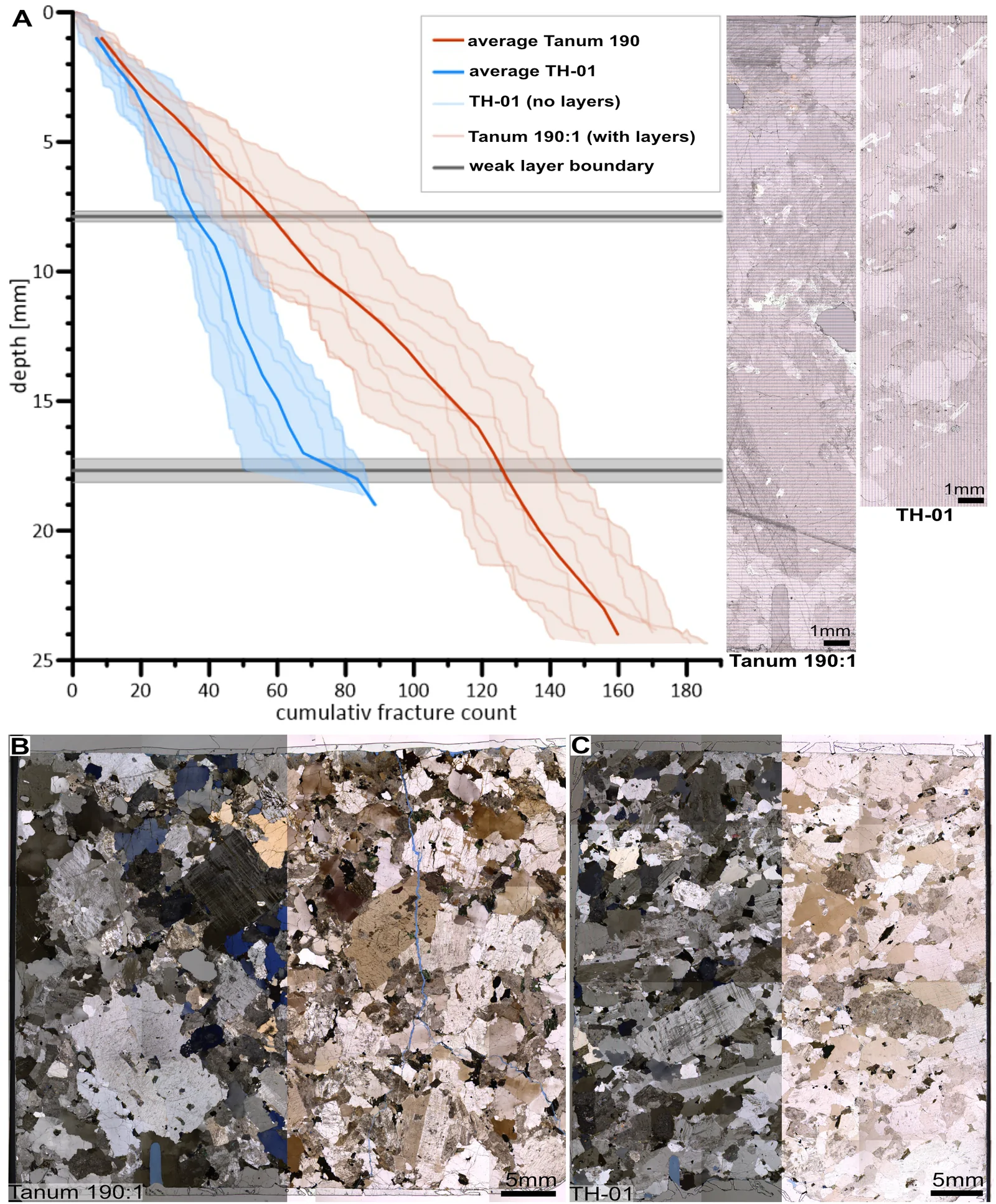
Microfracture count and thin sections from a carved (Tanum 190:1) and uncarved (TH-01) site. A: cumulative count of microfractures with depth along 8 evenly distributed lines, gray lines showing the depth of weak layers estimated for Tanum 190:1. Hich resolution scanned images used to count fractures. B: lambda (left) and plain polarized (right) image of thin section from Tanum 190:1. Weathering can be seen as altered minerals up to 2 mm and deeper along open fractures C lambda (left) and plain polarized (right) image of thin section from TH-01. Weathering appears only along open fractures.

Microfractures counted along 8 equally distributed lines in thin sections from Tanum 190:1 and TH-01 are shown in Fig 4A The cumulative fracture count increases significantly steeper at Tanum 190:1, where weak layers have been detected, than at the non-carved reference location TH-01 (no layers detected). This reflects a higher fracture density throughout. Individual counts show stronger increases at two depth levels between 6−11 mm and 15−20 mm surrounding the detected weak layers at 7.67 mm and 17.68 mm respectively. These increases are not observed in TH-01.

Observations from investigated outcrops (Fig 1), hand specimens (Fig 3) and thin sections (Fig 4B, C) show a regional homogenous structure of the Bohus granite. Thus, any changes in ultrasonic wave velocity cannot be explained by variations in rock texture and must be an indication of changing amount of microfractures [47].

### b) Ultrasonic depth estimation of layers

**Fig 5:**
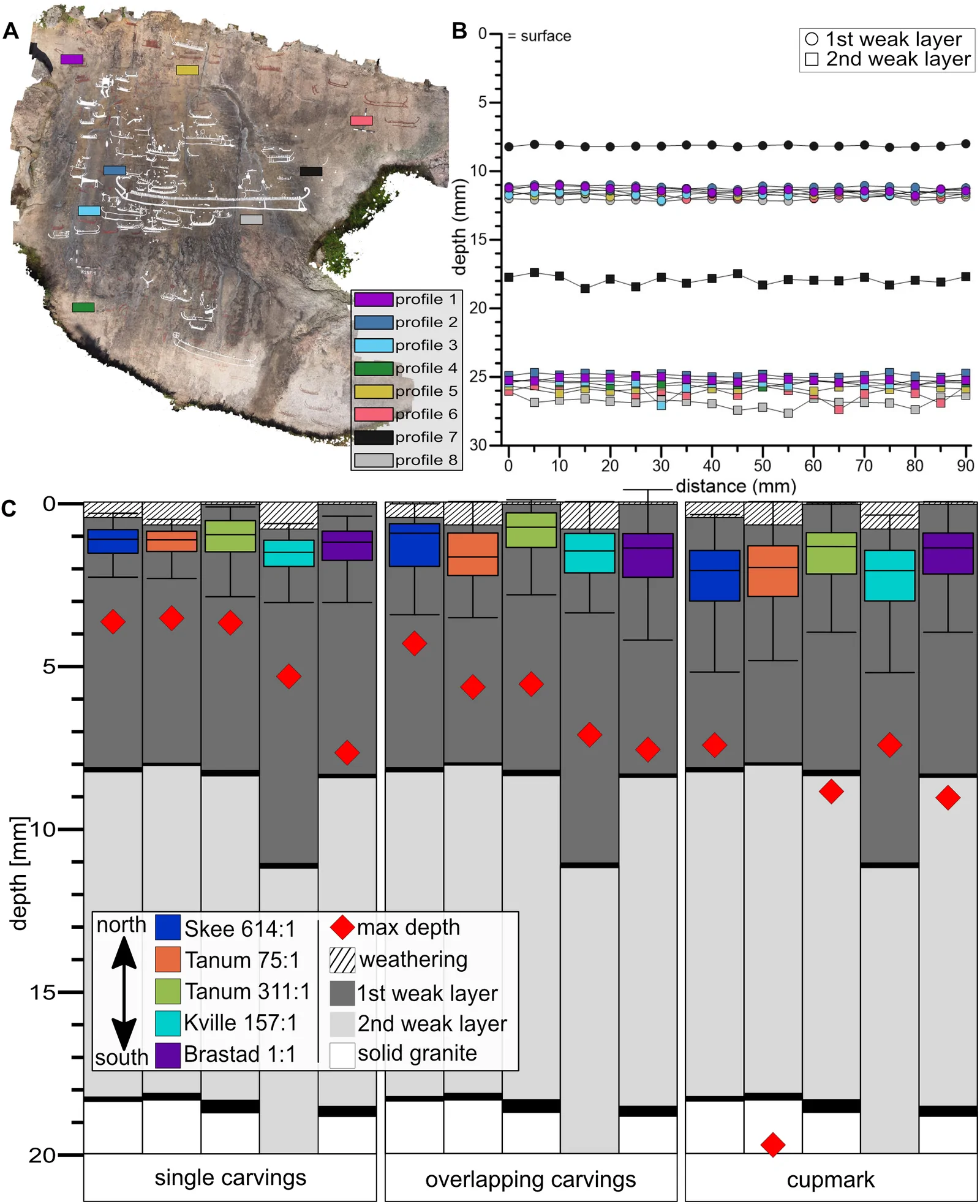
Results from B-scans and DEM-based depth measurements. A: location of profiles (B-scans) on Kville 157:1; Basemap picture from SHFA [48]; B: depth profiles of first and second reflective layer from all profiles shown in a; C: average depth of first and second layer at all sampling locations, and in comparison to carving depths and surface near weathering. Depth distribution shown as box-whisker plots based on the mean values of the deepest 10% of data points for each carved figure. Single carvings represent datapoints assigned to one figure, overlapping carvings represent datapoints assigned to more than one figure, cupmarks represent datapoints assigned to cupmarks. Figure modified after Liebl [49].

At all measured locations with carvings, reflective layers were found subparallel to the surface (Fig 5, Table 1). The depth of both reflective layers is constant at most locations (with reflective layer one at ~8–9 mm depth and layer two at ~18–19 mm, Table 1, Fig 3C). The only exception is Kville 157:1 where the first layer is at 11 mm depth and the second layer at 25 mm (Fig 5A, C). While most B-scans taken on all panels to determine the position of the reflective layers (see methods) are similar, Kville 157:1 is again an exception (Fig 5A, B). Profile 7 deviates from the others and shows a first layer depth of approximately 8.4 mm and 17.7 mm for the second layer, which is closer to the results of the other measured panels. In all cases the detected reflective layers are significantly deeper than the observed weathering depth and thus do not interfere with the signal.

### c) DEM-based depth calculations of carvings

Depth calculations for each panel are based on 19770–71798 grid points across the panels motifs (methods section). The average depth of individual carvings ranges from 0.96 mm on Tanum 311:1 to 1.52 mm on Kville 157:1 (Table 1, Fig 5C). The deepest carving in this study is a human figure found in Brastad 1:1 with up to 7.69 mm. Overlapping areas of two or more figures and cupmarks are evaluated separately. Except for the cupmarks, all analyzed motifs (single and overlapping carvings) do not exceed the first reflective layer (Fig 5C). There is a general trend of increased depth from North to South and in areas where two or more figures overlap (Fig 5C). Cupmarks are often deeper than other figures and can even exceed the depth of the weakened layers found (Fig 5C).

## Discussion

The rock surface we see today may in many cases be the same as the carvers saw during the Bronze Age. Protected by an amorphous coating layer created by the ice moving over the rock preventing deep weathering in the rock [33]. While this layer creates a visually pleasing canvas it is only few µm thick and does not have an impact on the overall rock strength, and therefore, on the possibility to carve on it. Post glacial weathering, on the other hand, has been suggested as a likely mechanism that allowed the carvers to use this extreme hard rock as a canvas for their art [19]. Swantesson [38] however, has shown that post-glacial weathering in the Bohus granite only affects less than a millimeter of the rock’s surface. Furthermore, at all locations in this study surface near weathering does not affect more than the uppermost 1 mm of the Bohus granite surface (Table 1, Figs 3, 5C) and thus does not interfere with the detected reflective layers. The weak layer can be systematically measured with similar depth across each outcrop indicating an overlying regional process as cause. Here, we identify this paraglacial weaking of the uppermost centimeter of the rock surface to be critical in preparation of a suitable canvas.

The weight of the overlaying ice during the last Ice Age applies a main principal stress in the direction of the normal force. The size of the applied stress changes with the thickness of the ice and is completely removed once the ice is molten. As a response to the applied stress and subsequent relief, surface-parallel microfractures are expected to form at a depth corresponding to the amount of load at a given point, thereby weakening the upper layer of the granite [50,51]. We can see this discontinuity at a depth of approximately 8–9 mm and 19–20 mm in the sample from Tanum 190:1 (Fig 3B), which agrees with the depth of reflective layers we found in most B-scan profiles taken with ultrasonic wave measurements (Fig 5C, Table 1). Depth modelling of all carvings on the selected panels show that the carvers have used the first reflective layer for their motifs only (Fig 5C). While one motif at Brastad 1:1 is close to the weak layer boundary not a single carving reaches out of the reflective layer or into the unweakened rock beneath.

In contrast, the completely uncarved sample from location TH-01 does not show these reflective layers (Fig 1, insert) even though it has a perfectly polished surface and is inside an area with very high carving density. Other work has presented a range of factors for choosing a rock as canvas such as quality and smoothness, cracks and depressions, shape, minilanscapes, slope, orientation, amount, intensity, and direction of sunlight, proximity to water, waterflows, proximity to settlements, graves or pathways (e.g., [2,4,5,7,52–59]).We argue that the existence of synglacial weakening related reflected layer is an additional major factor for the rock surface to be carved.

Regarding Kville 157:1 the slope around profile 7 shows no motifs (Fig 5A, black profile), despite the occurrence of a first reflective layer at ~ 8 mm depth. Profile 7 was taken on part of the panel that has a slightly different orientation and is a bit steeper compared to the remaining rock panel. The steeper surface reduces the impact of the normal force from the ice load and thus decreases the resulting stress applied to the surface compared to the remaining carved panel (Fig 5A). Here, a first reflected layer is observed at ~11 mm (Fig 5, Table 1) indicating a correlation between the intensity of weakening the rock surface and the occurrence of carvings. The lack of carvings at location TH-01 and the preferred placement on surfaces with deeper reflective layers suggest that the carvers were able to not only detect the existence of these layers but were able to distinguish their relative depth as a canvas quality marker. Making the placement of motifs a decision based on geological properties rather than a purely artistic choice.

The existence of reflective layers is not visible at the surface of the rock. It takes knowledge about the rocks properties to distinguish suitable rock panels from less- or non-suitable ones. Even more expertise is necessary to compare the quality of the potential canvas as the difference is minimal. We used ultrasonic measurements, which are based on the velocity with which a sound wave travels through the rock, to determine the quality of the rock surface for each location. Following this concept and using a wavelength within a range humans can hear, the different velocities based on the rock quality are expected to result in slight variations of sound [60]. Although this needs further investigation, it might be possible with enough training and expertise to locate “good” and “bad” placements by, e.g., knocking on the rock or moving a metal object across the surface. Much like metal workers relying on eyesight to distinguish colors to estimate the heat in furnaces [61] or modern geologists do quick quality checks on rocks during geological surveys by knocking on them [60,62], we suggest the possibility that rock carvers may have relied on training their senses, in this case hearing to distinguish the sound of a rock that is suitable for carving and such that are not. Making a conscious evaluation like this based on properties found in the rock can be considered geological knowledge even if the processes behind were not understood. It is important to distinguish between geological understanding of processes and knowledge from experience [63]. An example is the oldest known geological map from 1150 BC [64]. Producing it needed the geological knowledge to differentiate the rocks, and describe and locate them in the landscape. Accumulation of knowledge and further refinement is part of human nature, propagating and improving the knowledge about how to find a good canvas over time [65,66].

Areas with overlapping carvings are deeper than individual figures indicating that the depth was increased during the reworking of the original motif while creating a second figure. In addition, a weak trend seems to exist of increasing maximum depth from Skee 614:1 in the north to Brastad in the south. There is no connection with grain sizes, fabric, or the chemistry of the rocks found that could explain such a trend implying a non-geological reason. Further investigation is needed to see if the trend persists for a larger number of locations.

Cupmarks reach the greatest depth and often exceed the first and in one case even the second weakened layer. This exceptional depth and the very smooth surface inside the cupmarks are a strong indicator for the use of a different technique compared to other figurative carvings. The depths could, e.g., be achieved through long grinding with sand.

## Conclusion

An uppermost 8–12 mm thick layer below the surface of the Bohus granite represents a rheologically weaker structure, which is caused by stress release after the last glacial. All investigated carvings except of cupmarks are fully placed inside this weaker layer. In case it is less well developed or missing no carvings are present. Therefore, it is paraglacial surface weaking of the Bohus granite, which has a geological impact on whether a rock panel was carved, and not postglacial weathering, which in most places does not affect the rock deep enough

The interplay of paraglacial stress relief and surface topography determines the quality of the granite as possible canvas. The choice of placement made by the carvers correlates with the quality of the rock as canvas as determined by ultrasonic measurements. This is a strong argument for existing knowledge about rock properties as these parameters cannot be seen with a quick look at the surface.

The digital elevation model (DEM) depth modelling of large datasets reveals a geographical change in depth of the carved motifs from North to South of Bohuslän in western Sweden. This is not related to rock properties of the Bohus granite and, therefore, argues for a non-geological reason.

Cupmarks are statistically deeper than all other motifs and partly reach into a second weak layer or even the solid bedrock below the two weaker, surface near zones. This either indicates the use of a different production method, different age or multiple reworking of these structures.

## Supporting information

S1 File Placement and B-scan profiles. Placement of b-scan profiles on each rock art panel and reference location including determined depth for each profile.
(PDF)
