# Supplementary material for: Rock carving depth reflects carvers’ geological insight
Source: PLoS One. 2026 Sep 21;21(9):e0358075. doi: 10.1371/journal.pone.0358075 (PMC13592626; doi:10.1371/journal.pone.0358075)
Supplement: Supplementary file 1 [file pone.0358075.s001.pdf]

TH-01

no layer

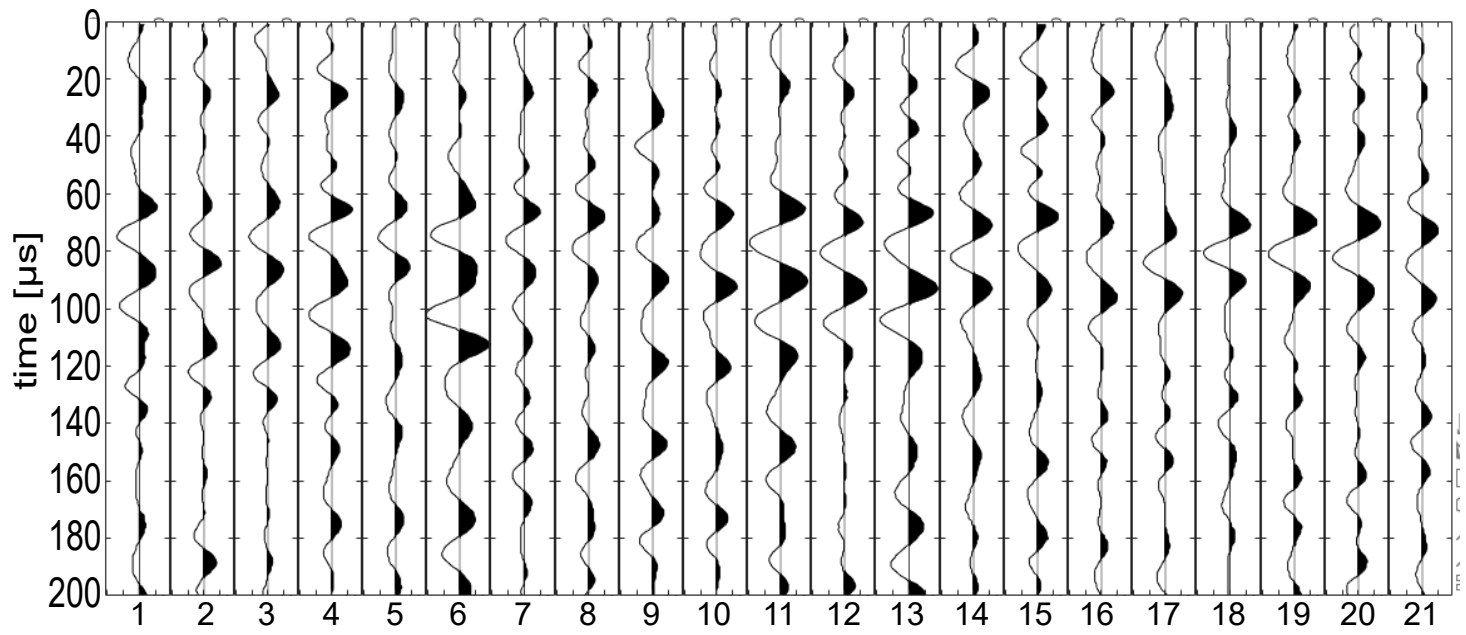

Profile 1
orientation
240/06
velocity
2345m/s
no layer

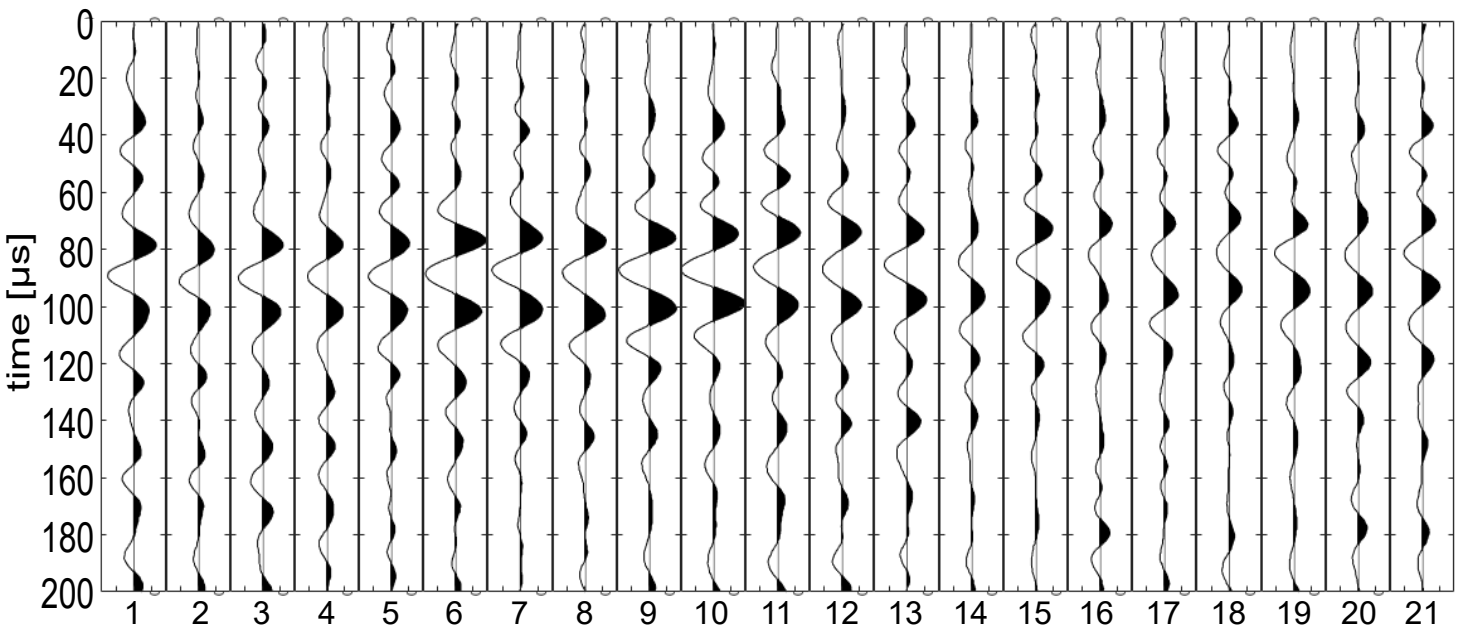

Profile 2
orientation
234/09
velocity
2345m/s
no layer

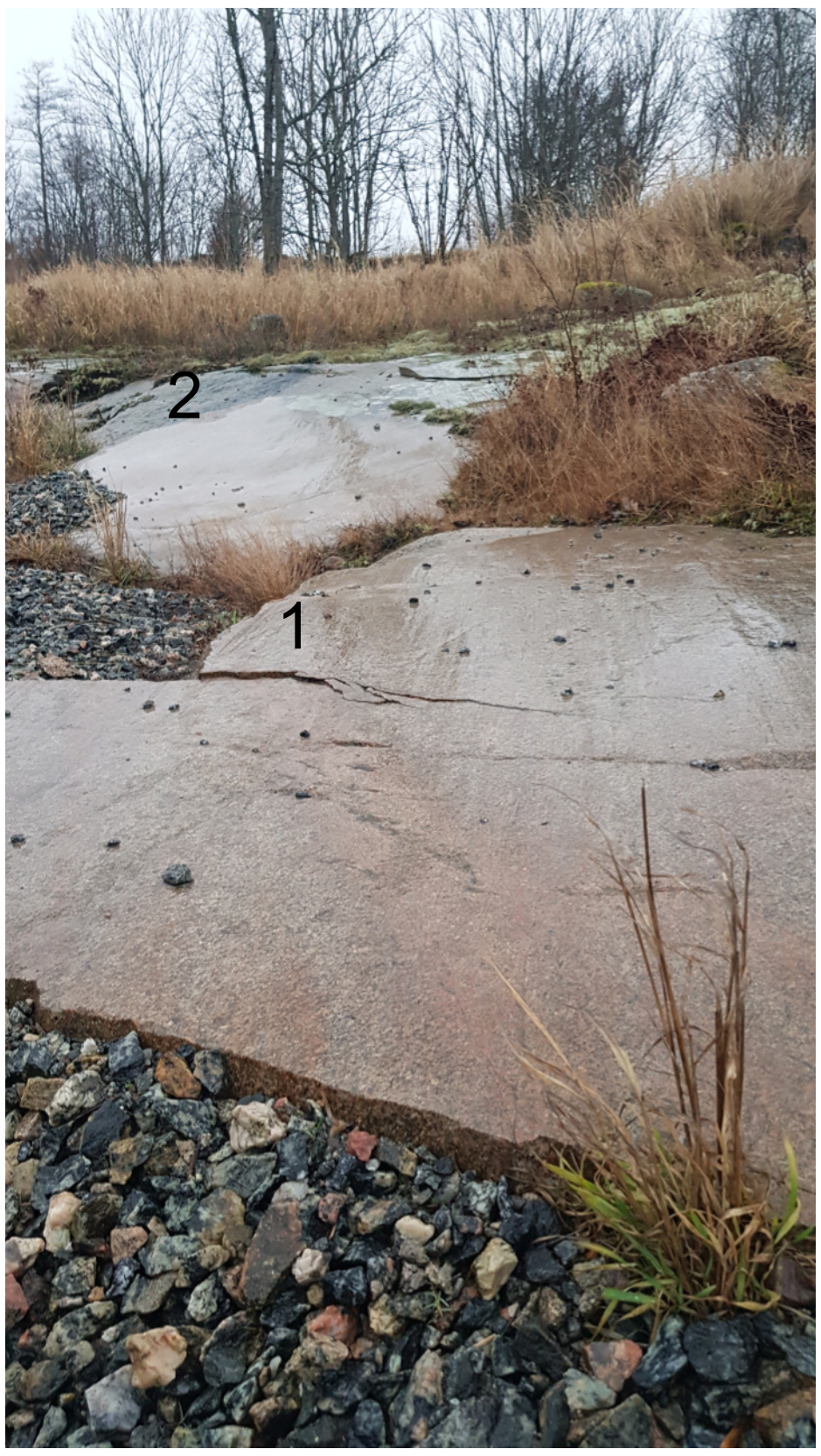

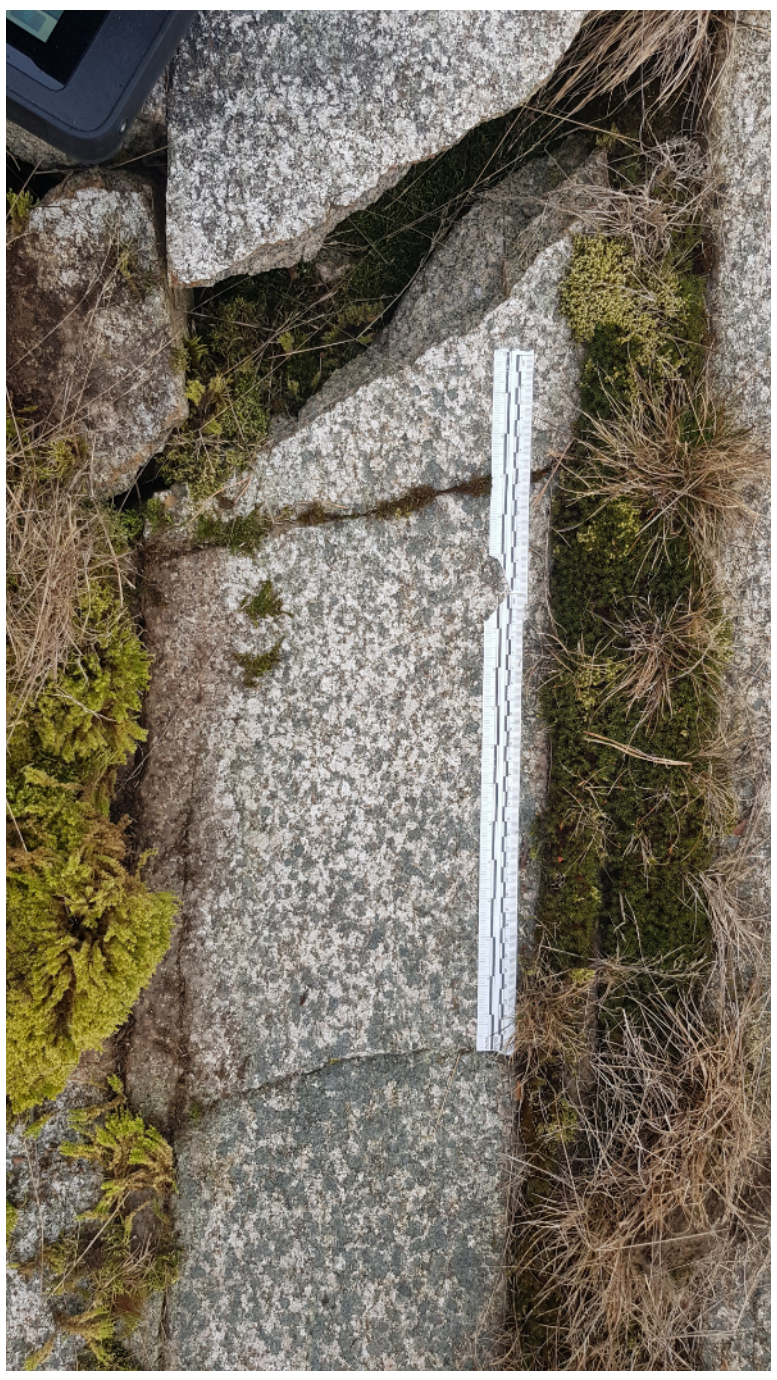

Tanum 190:1

1st layer
7.87mm±0.02mm
2nd layer
17.68mm±0.46mm

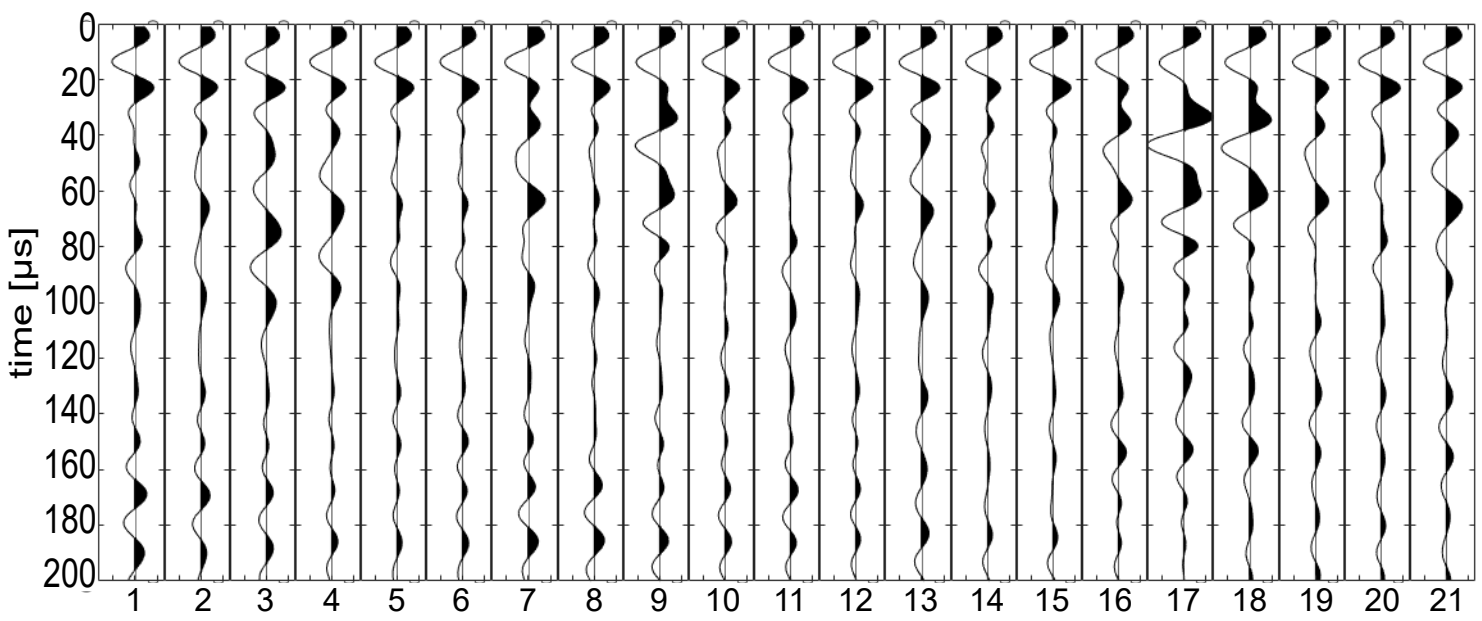

Profile 1

velocity
1869 m/s
1st layer
7.87mm±0.20mm
2nd layer:
17.68mm±0.46mm

Skee 614:1

1st layer average
8.22mm±0.08mm
2nd layer average
18.29mm±0.17mm

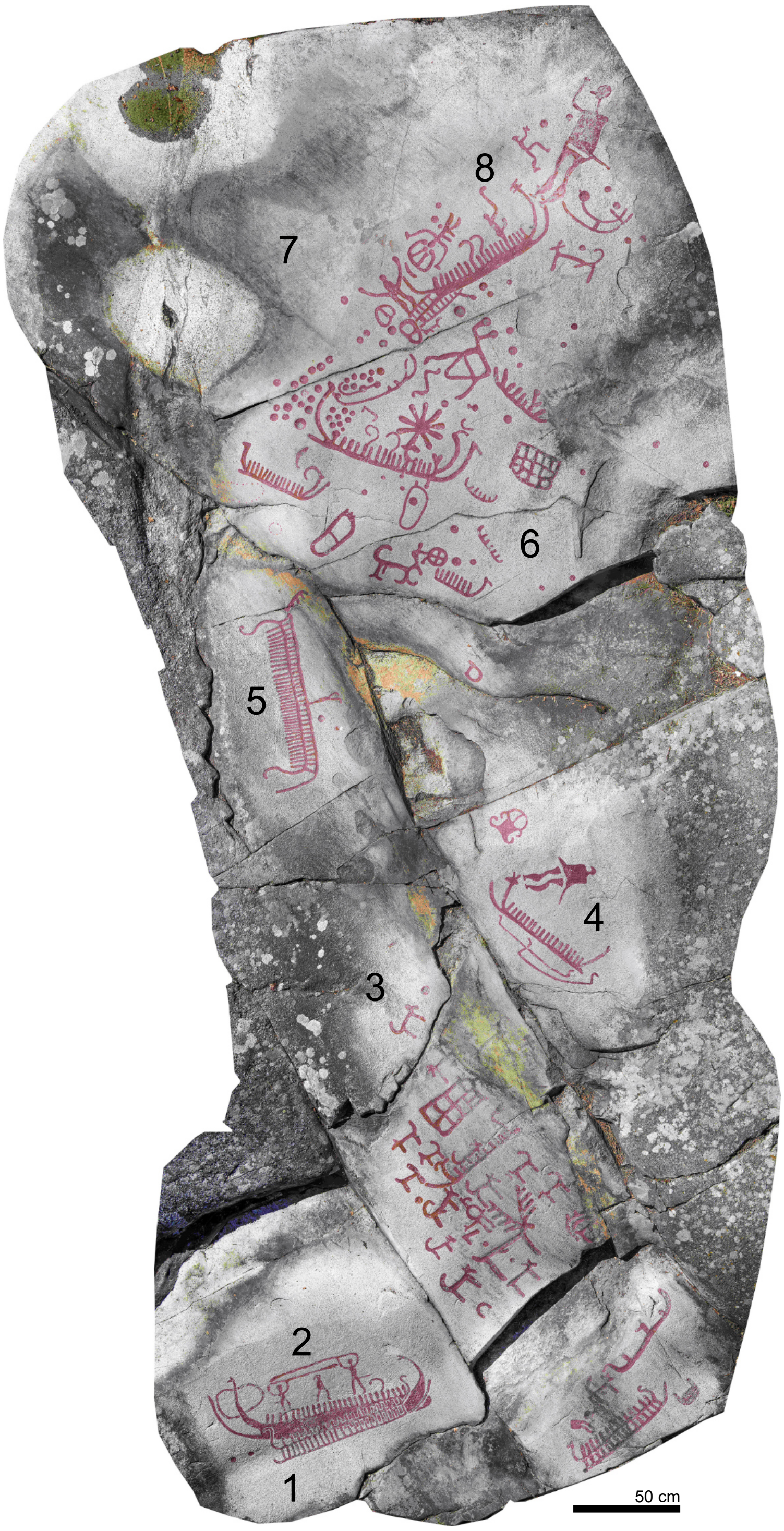

photo: Moyano Di Carlo, Julián. (2022). Ortofoto (SfM) av L1968:5160, SHFA, åtkomst 8 July 2026 på https://shfa.dh.gu.se/image/135299

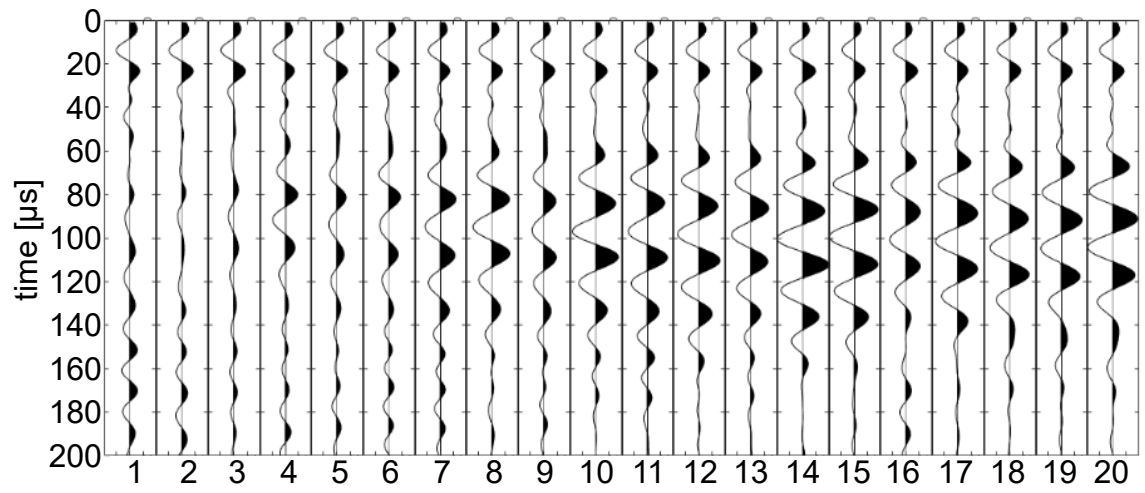

Profile 1
orientation
239/14
velocity
1928 m/s
1st layer
8.16mm±0.22mm
2nd layer:
18.09mm±0.48mm

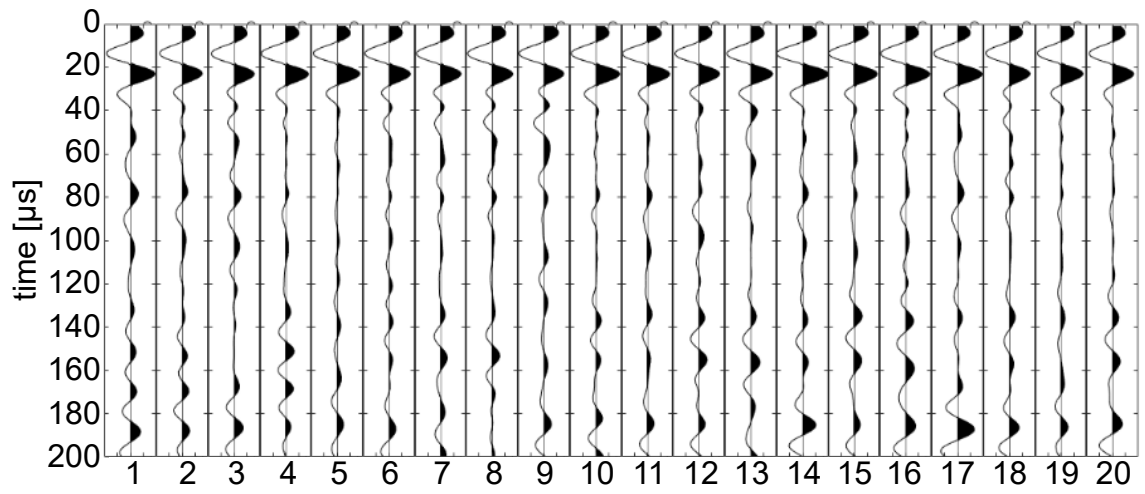

Profile 2
orientation
310/23
velocity
1928 m/s
1st layer
8.00mm±0.21mm
2nd layer:
17.86mm±0.48mm

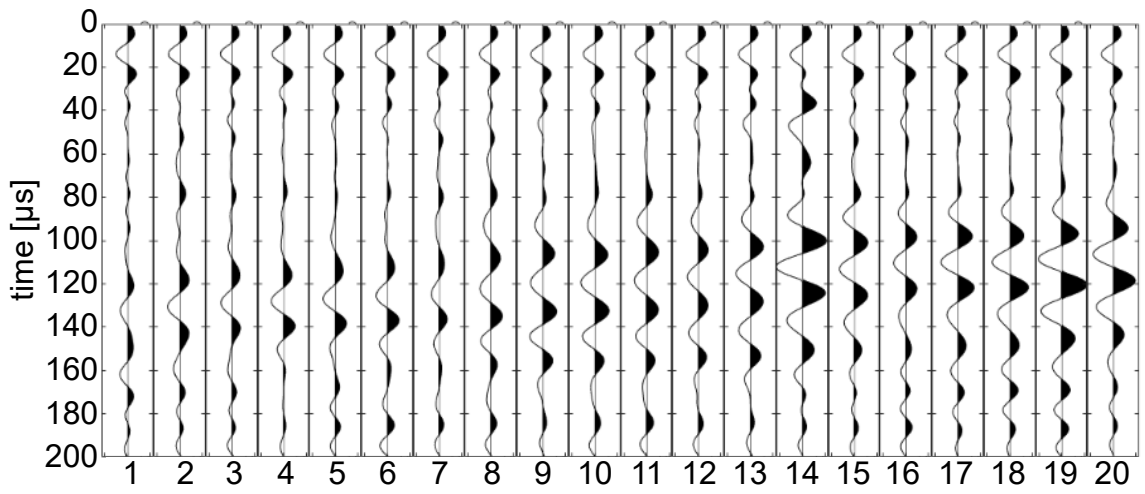

Profile 3
orientation
069/14
velocity
1928 m/s
1st layer
8.34mm±0.22mm
2nd layer:
18.29mm±0.49mm

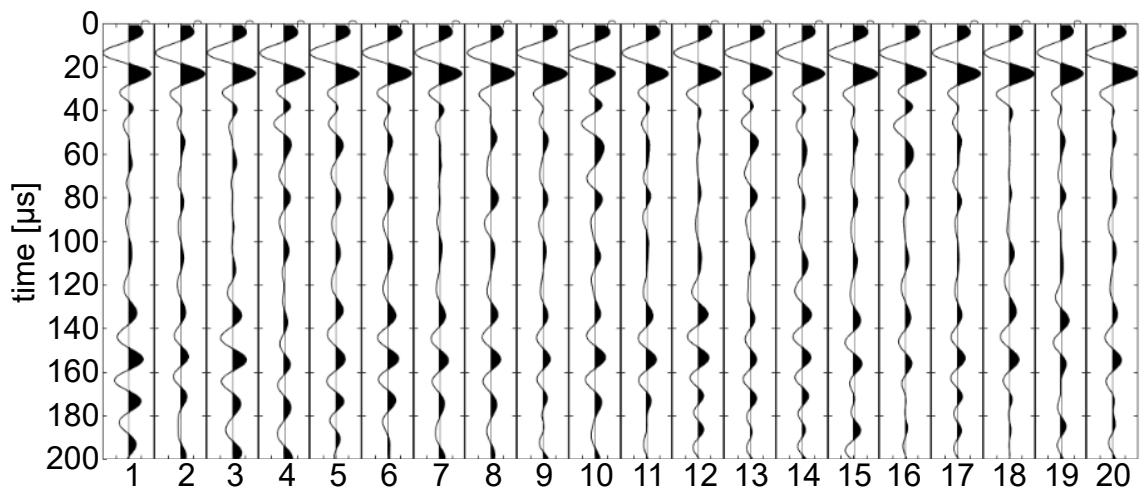

Profile 4
orientation
100/25
velocity
1928 m/s
1st layer
7.99mm±0.21mm
2nd layer:
17.89mm±0.48mm

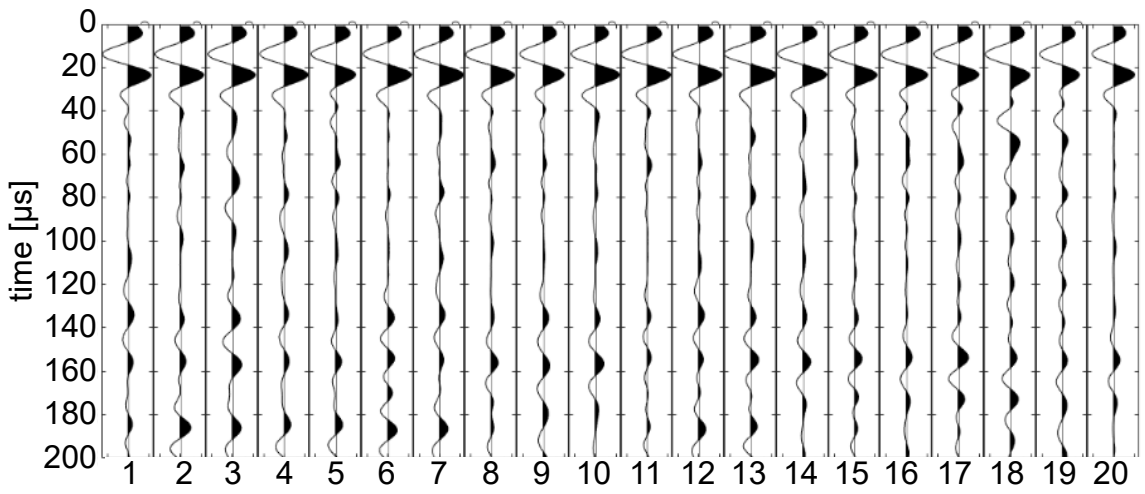

Profile 5
orientation
004/18
velocity
1928 m/s
1st layer
8.17mm±0.22mm
2nd layer:
18.67mm±0.50mm

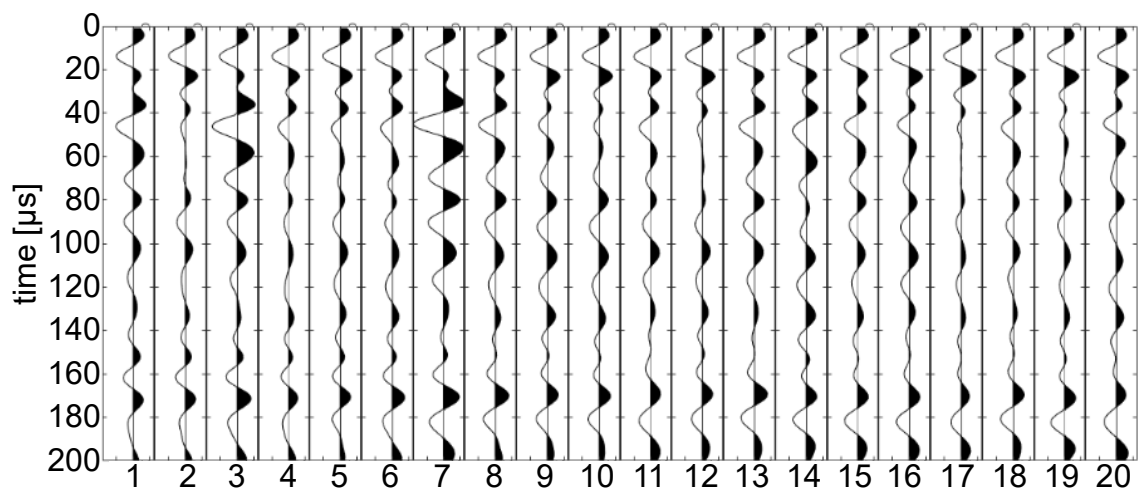

Profile 6
orientation
104/12
velocity
1928 m/s
1st layer
8.28mm±0.22mm
2nd layer:
18.35mm±0.49mm

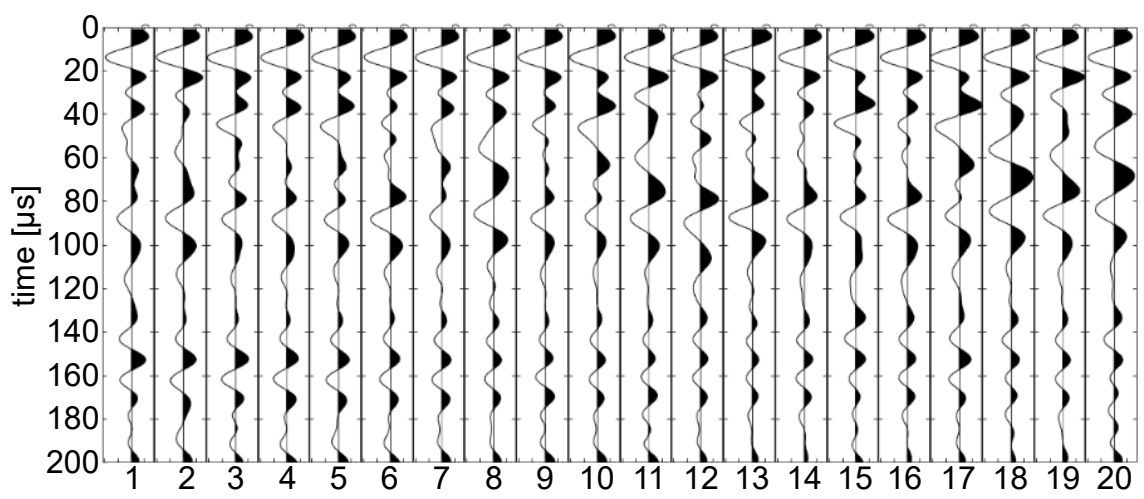

Profile 7
orientation
134/09
velocity
1928 m/s
1st layer
8.41mm±0.22mm
2nd layer:
18.50mm±0.49mm

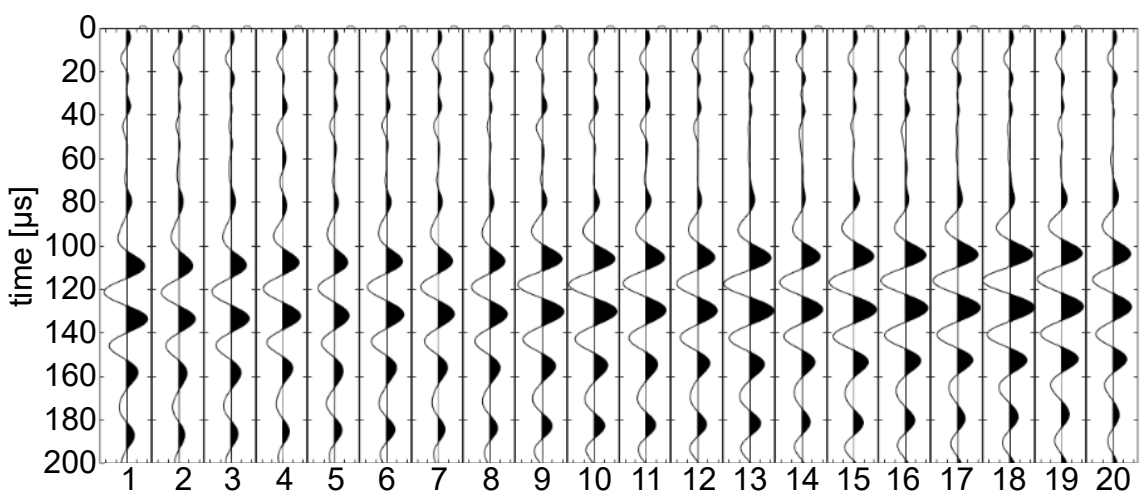

Profile 8
orientation
107/04
velocity
1928 m/s
1st layer
8.39mm±0.22mm
2nd layer:
18.69mm±0.50mm

Tanum 75:1

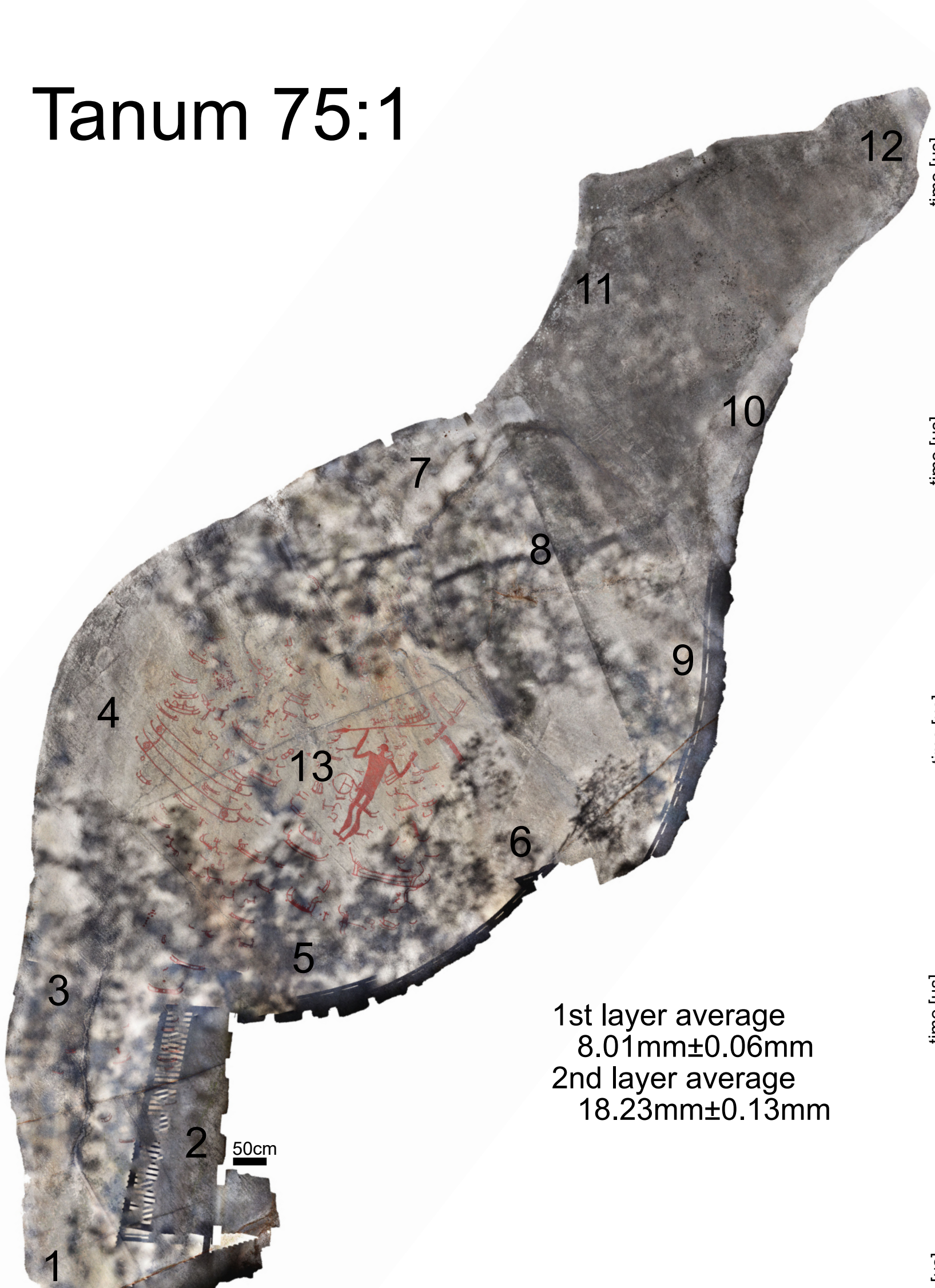

1st layer average
8.01mm±0.06mm
2nd layer average
18.23mm±0.13mm

photo: Horn, Christian och Potter, Rich. (2022). Ortofoto (SfM) av L1968:7559, SHFA, åtkomst 8 July 2026 på https://shfa.dh.gu.se/image/135270

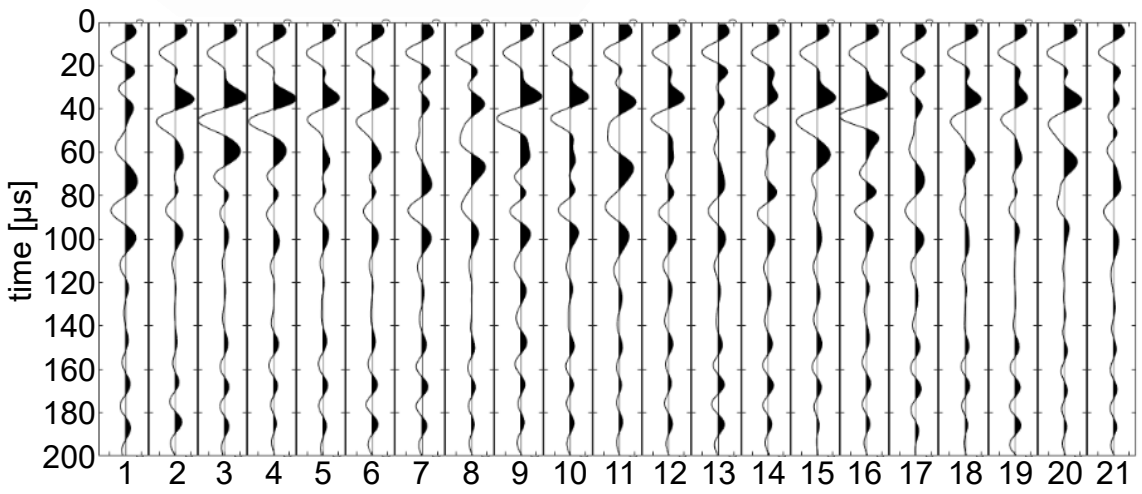

Profile 1
orientation

velocity
1883 m/s
1st layer
8.30mm±0.22mm
2nd layer:
19.29mm±0.50mm

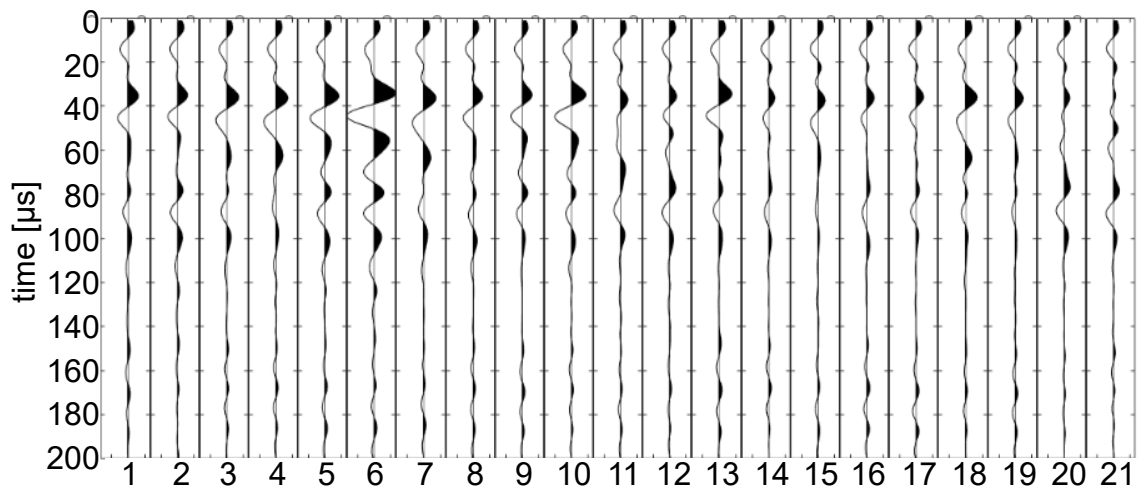

Profile 2
orientation

velocity
1858 m/s
1st layer
8.24mm±0.21mm
2nd layer:
21.19mm±0.56mm

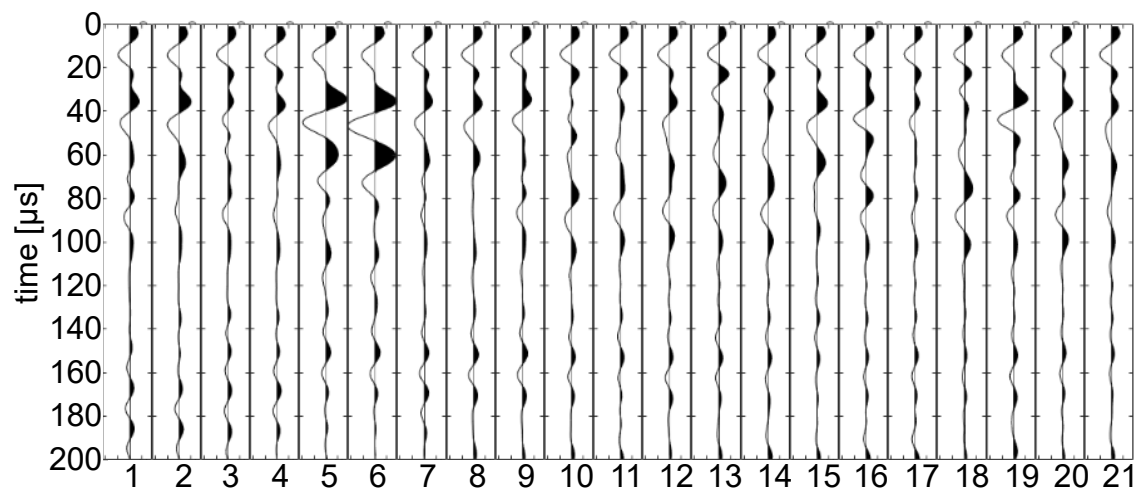

Profile 3
orientation

velocity
1880 m/s
1st layer
8.22mm±0.21mm
2nd layer:
19.03mm±0.50mm

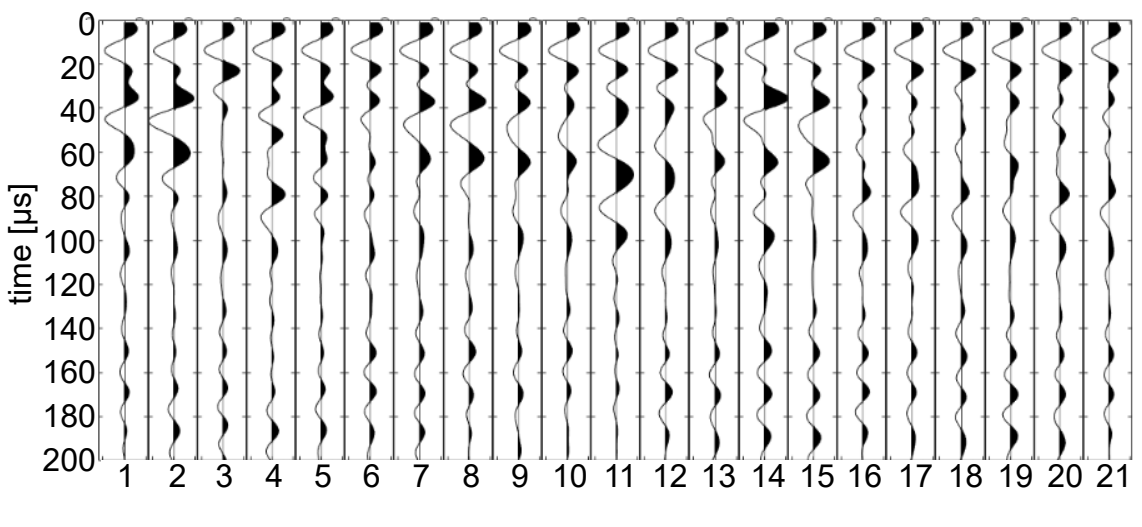

Profile 4
orientation

velocity
1875 m/s
1st layer
8.03mm±0.21mm
2nd layer:
17.88mm±0.46mm

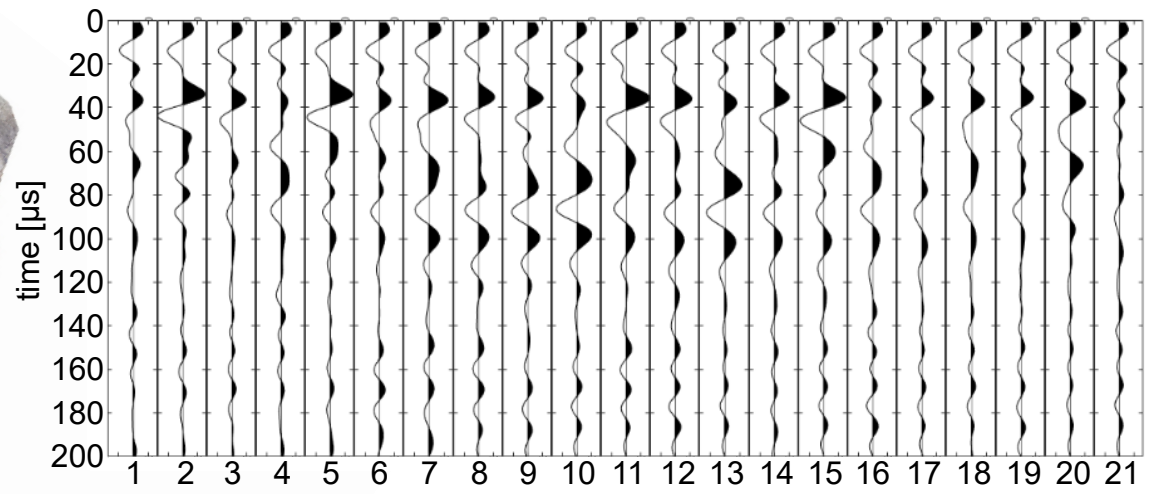

Profile 5
orientation

velocity
1848 m/s
1st layer
8.81mm±0.24mm
2nd layer:
20.44mm±0.54mm

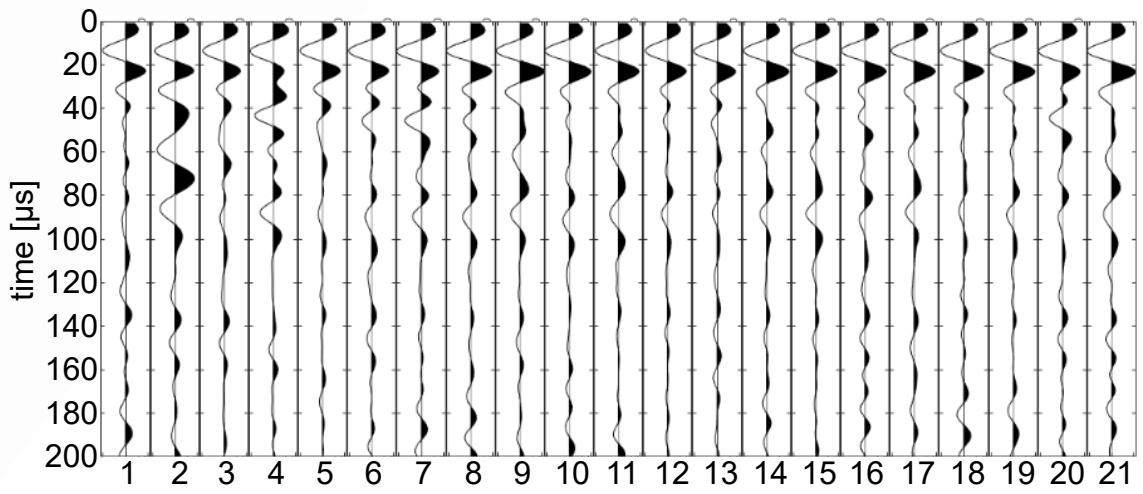

Profile 6
orientation

velocity
1816 m/s
1st layer
7.66mm±0.20mm
2nd layer:
16.94mm±0.44mm

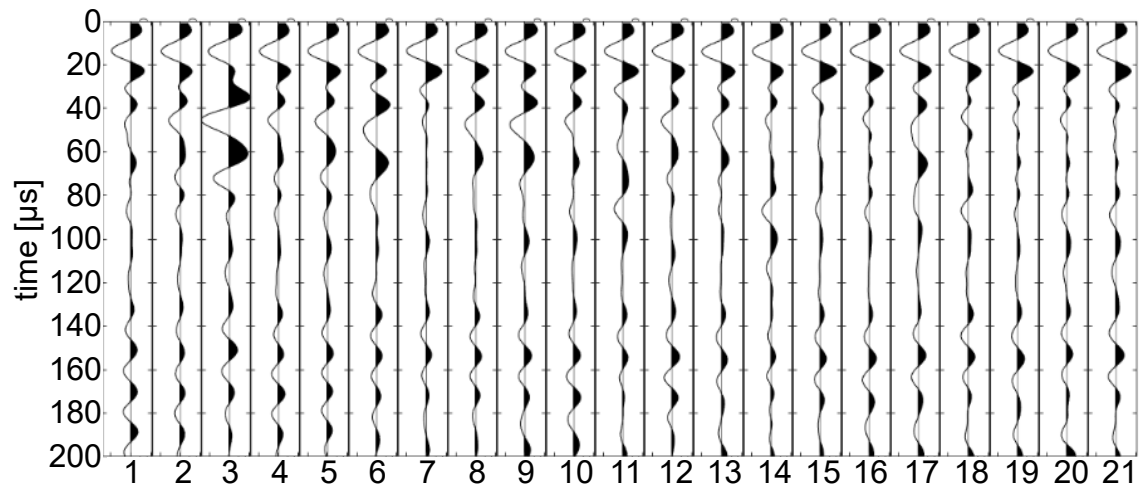

Profile 7
orientation

velocity
1842m/s
1st layer
7.82mm±0.20mm
2nd layer:
17.42mm±0.45mm

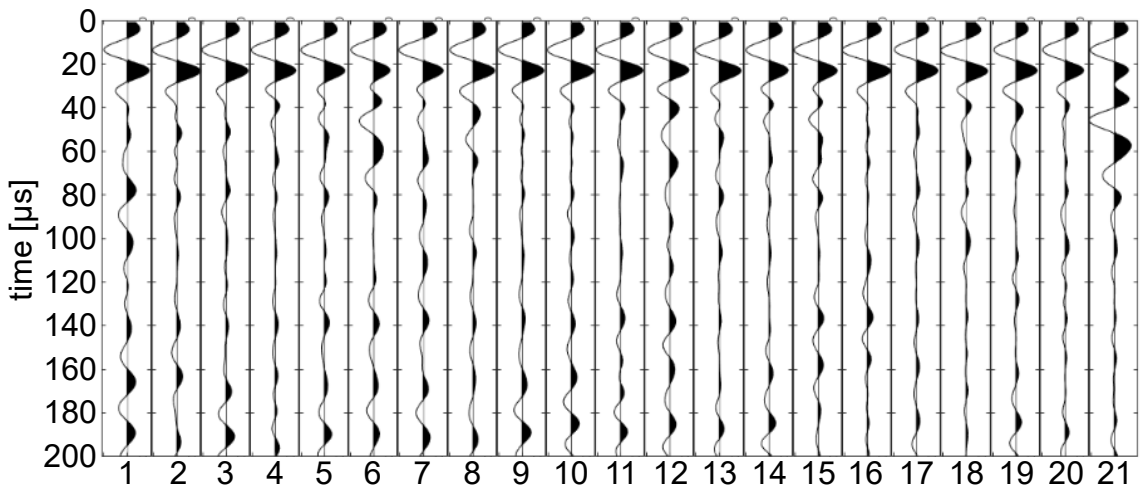

Profile 8
orientation

velocity
1842m/s
1st layer
7.72mm±0.20mm
2nd layer:
17.16mm±0.45mm

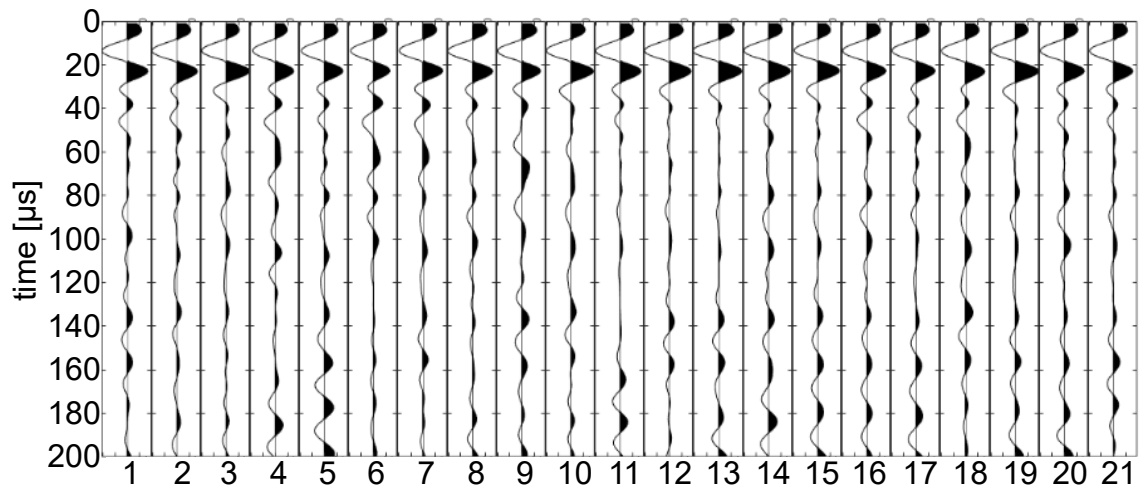

Profile 9
orientation

velocity
1842 m/s
1st layer
7.67mm±0.20mm
2nd layer:
17.06mm±0.44mm

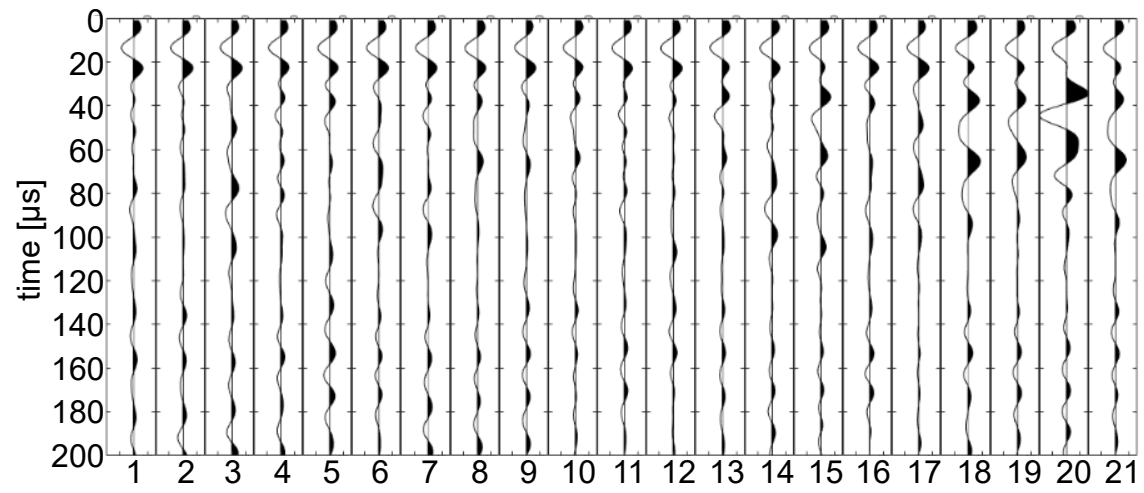

Profile 10
orientation

velocity
1886 m/s
1st layer
7.94mm±0.21mm
2nd layer:
17.75mm±0.46mm

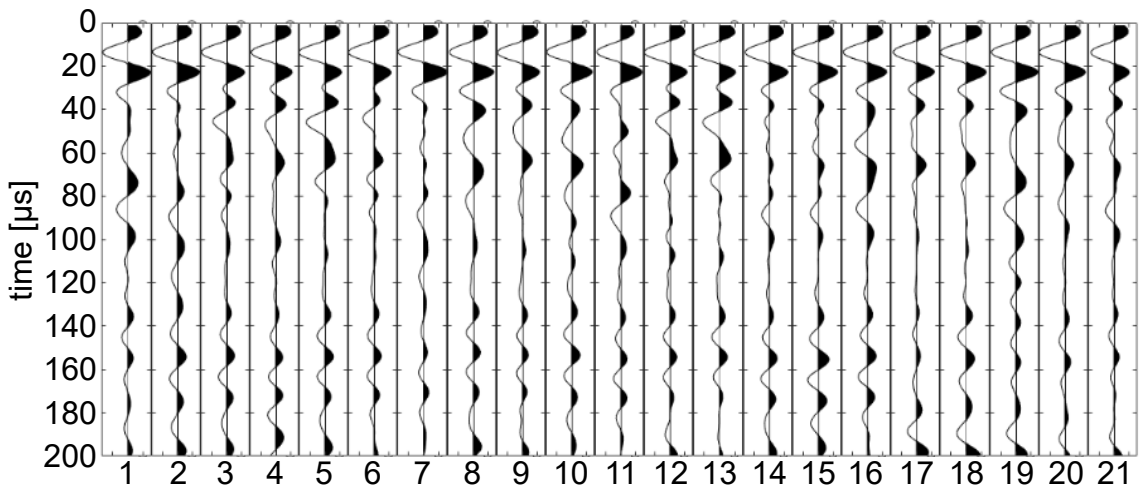

Profile 11
orientation

velocity
1869 m/s
1st layer
7.74mm±0.20mm
2nd layer:
17.28mm±0.45mm

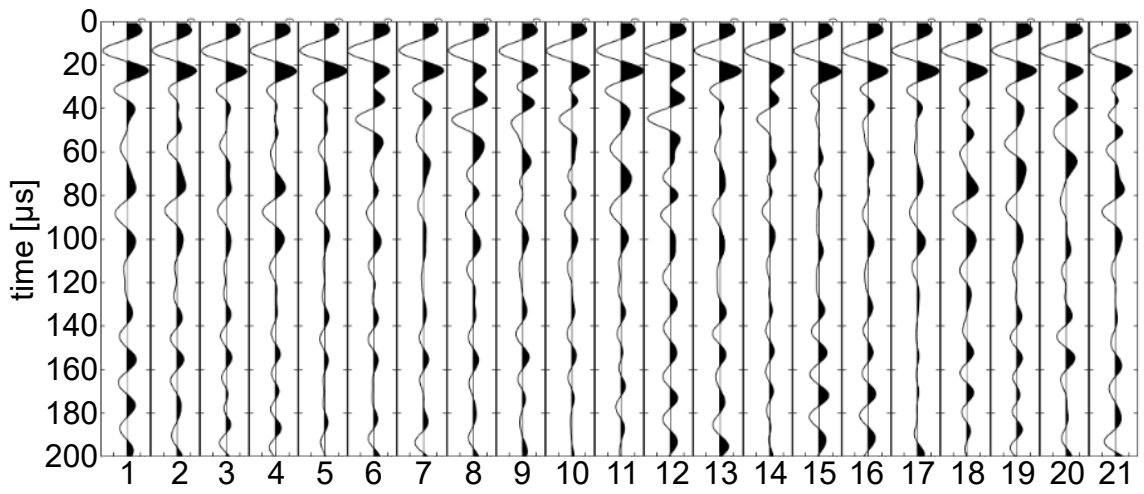

Profile 12
orientation

velocity
1878m/s
1st layer
7.87mm±0.20mm
2nd layer:
17.53mm±0.45mm

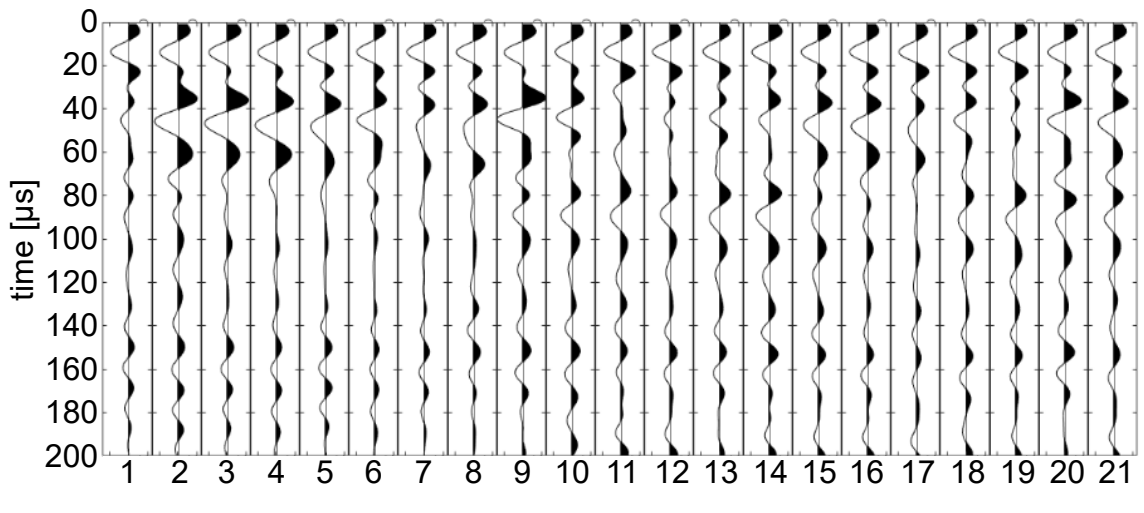

Profile 13
orientation

velocity
1874m/s
1st layer
8.07mm±0.21mm
2nd layer:
18.04mm±0.47mm

Tanum 311:1

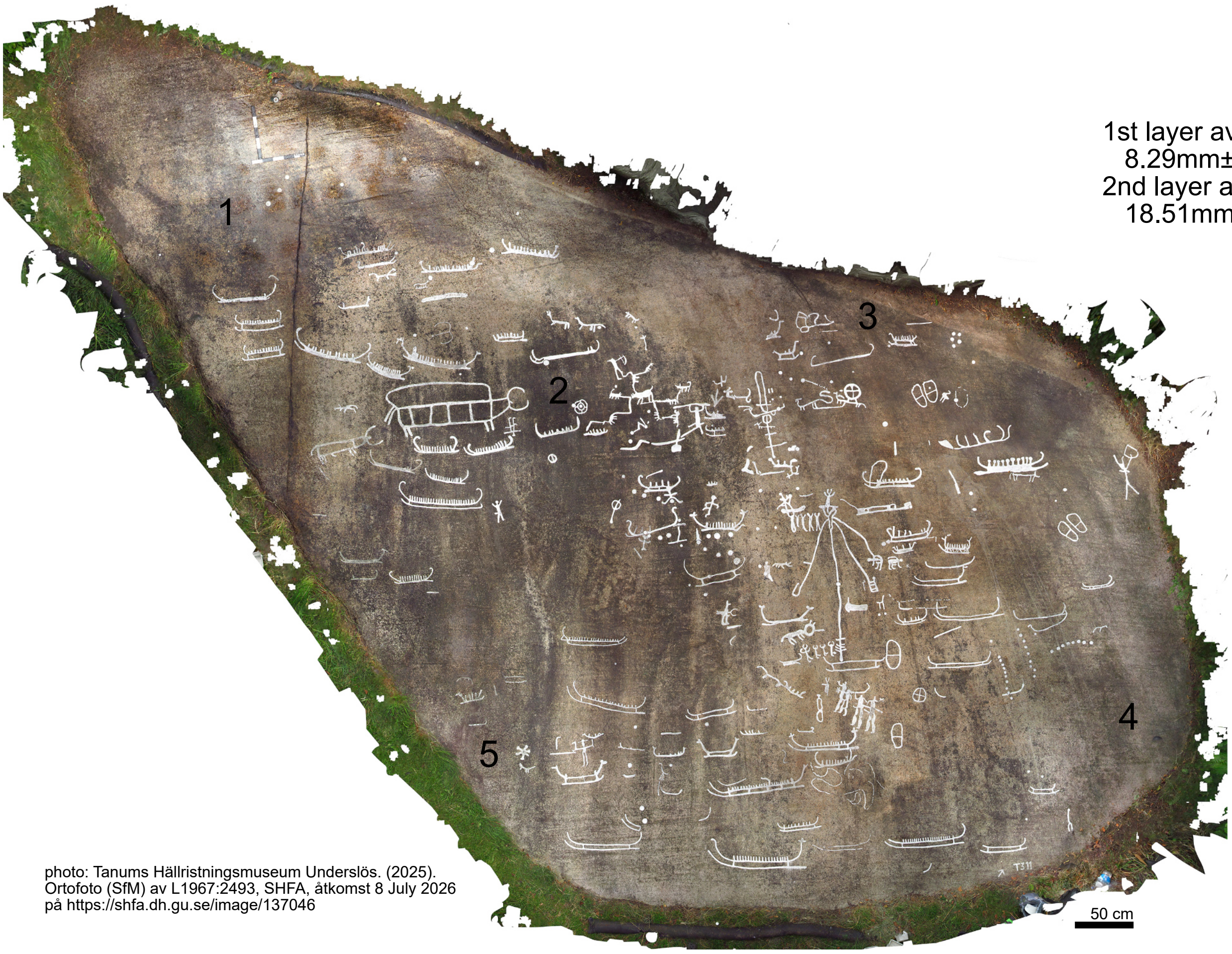

1st layer average
8.29mm±0.10mm
2nd layer average
18.51mm±0.21mm

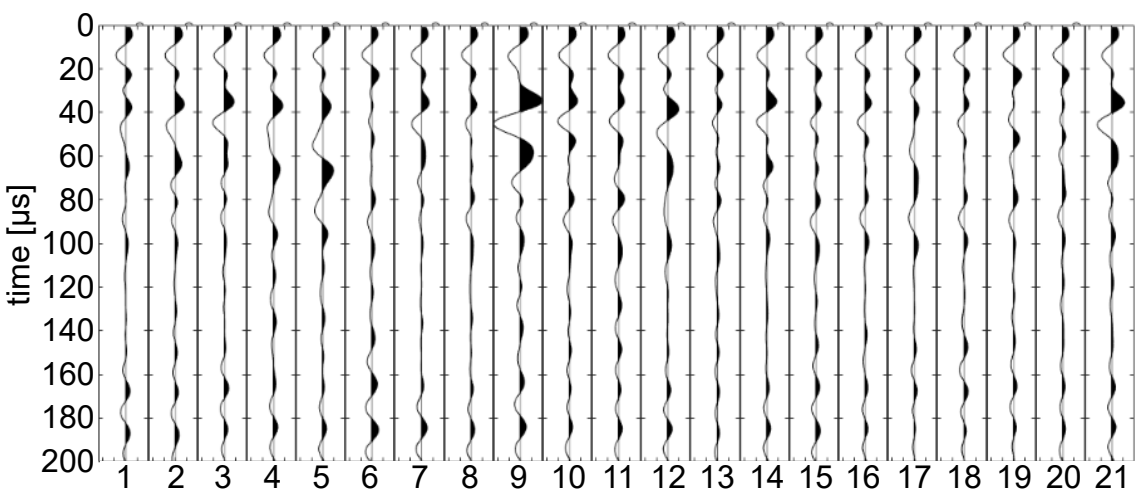

Profile 1
orientation

velocity
1842 m/s
1st layer
7.93mm±0.21mm
2nd layer:
18.06mm±0.47mm

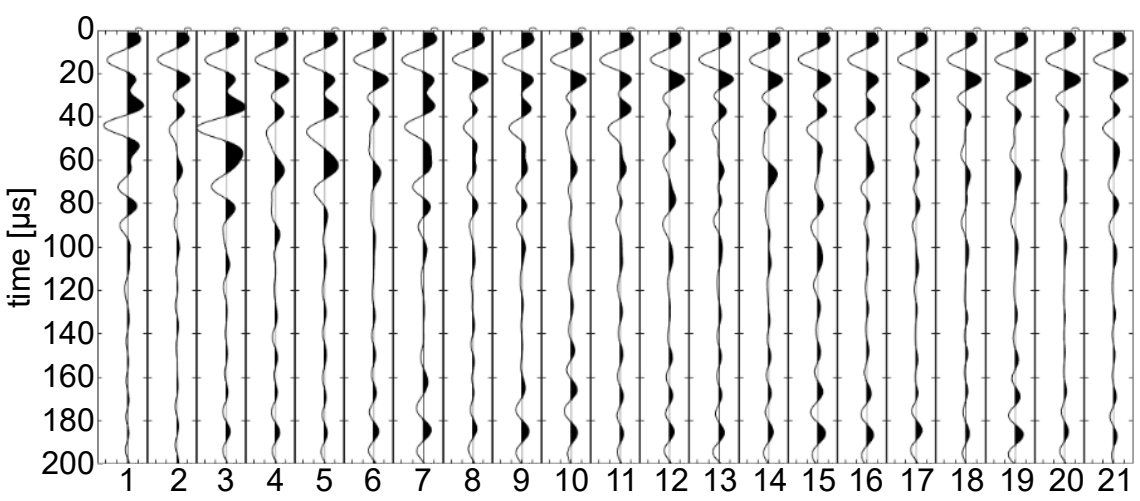

Profile 2
orientation

velocity
1837 m/s
1st layer
7.76mm±0.20mm
2nd layer:
17.27mm±0.45mm

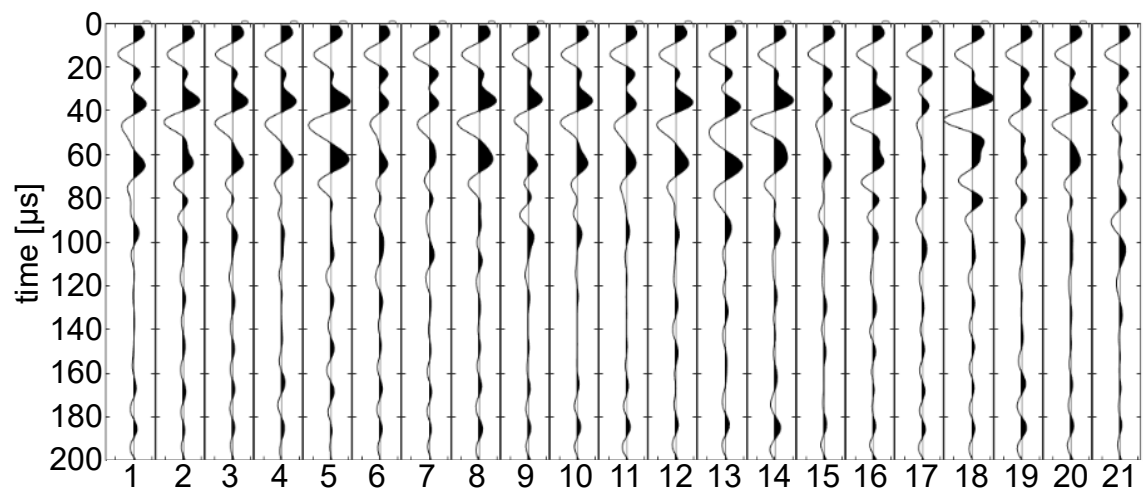

Profile 3
orientation

velocity
1851 m/s
1st layer
8.32mm±0.22mm
2nd layer:
18.61mm±0.48mm

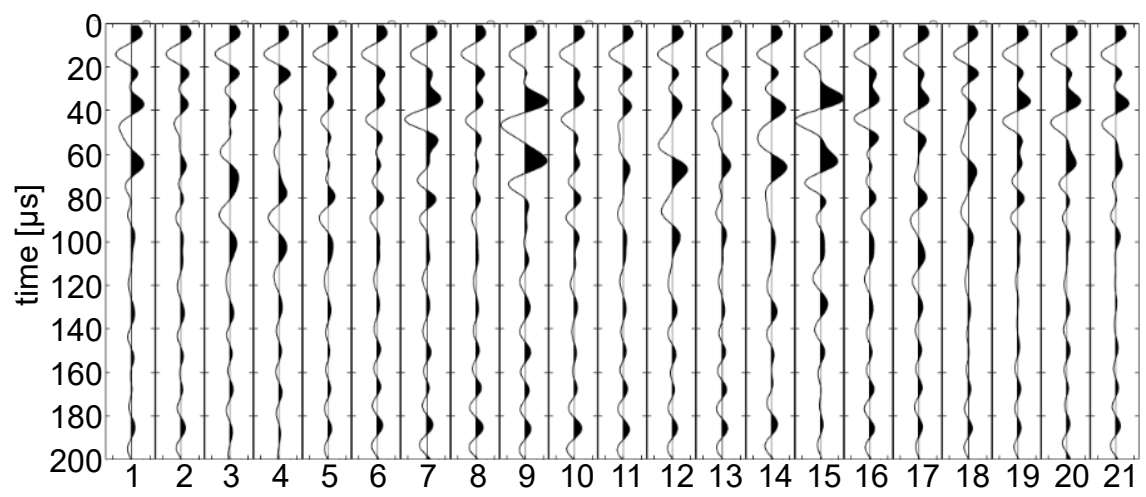

Profile 4
orientation

velocity
2018m/s
1st layer
9.12mm±0.24mm
2nd layer:
20.16mm±0.52mm

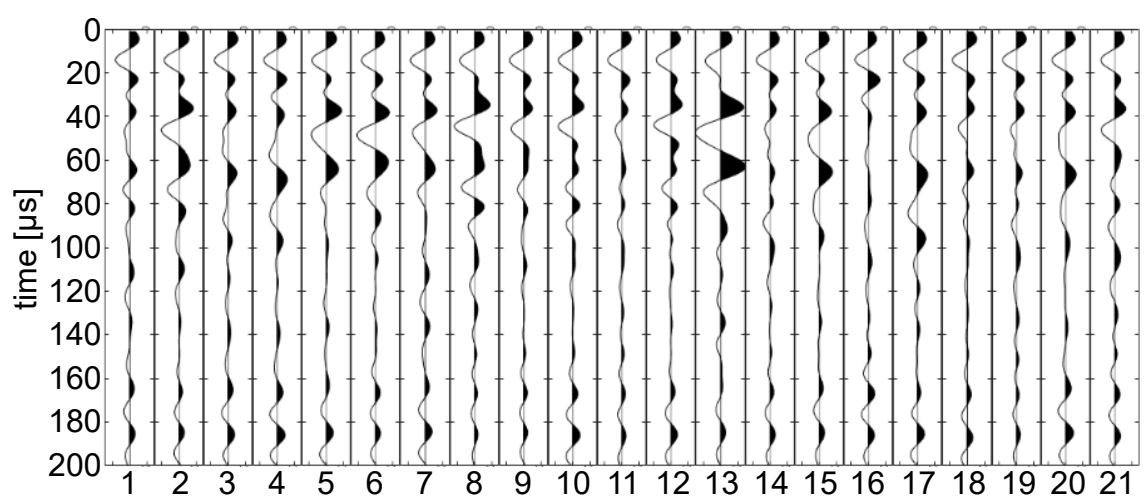

Profile 5
orientation

velocity
1837 m/s
1st layer
8.29mm±0.22mm
2nd layer:
18.43mm±0.48mm

Kville 157:1

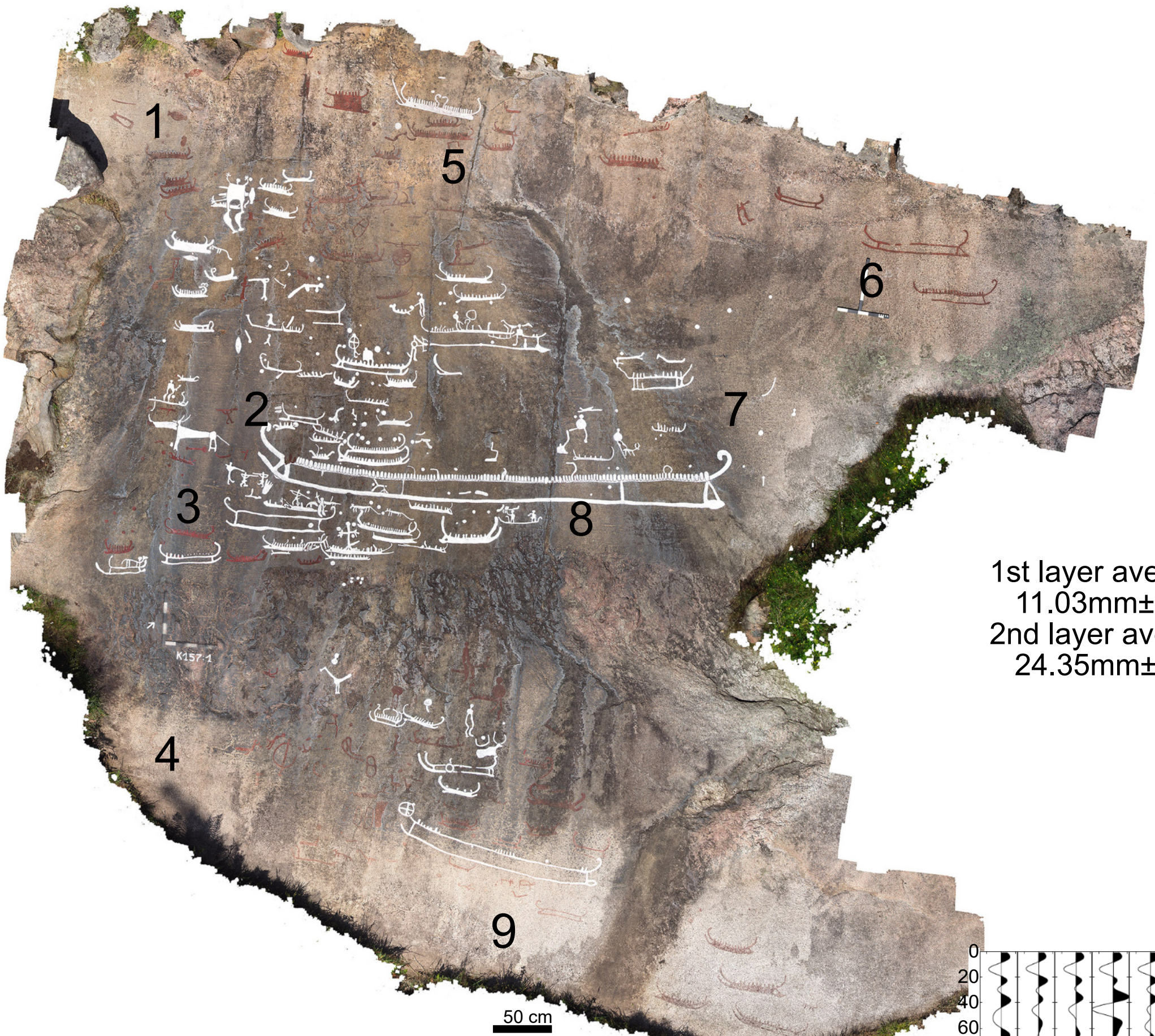

Tanums Hällristningsmuseum Underslös. (2022). Ortofoto (SfM) av L1969:5872, SHFA, åtkomst 8 July 2026 på https://shfa.dh.gu.se/image/133409

1st layer average
11.03mm±0.10mm
2nd layer average
24.35mm±0.22mm

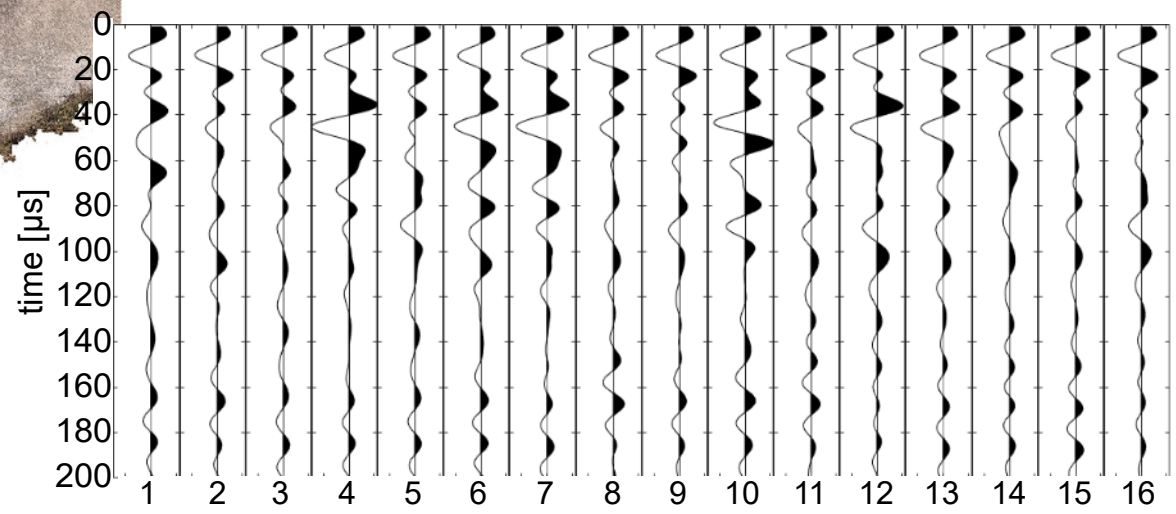

Profile 5
orientation
154/08
velocity
2744 m/s
1st layer
11.63mm±0.34mm
2nd layer:
25.82mm±0.73mm

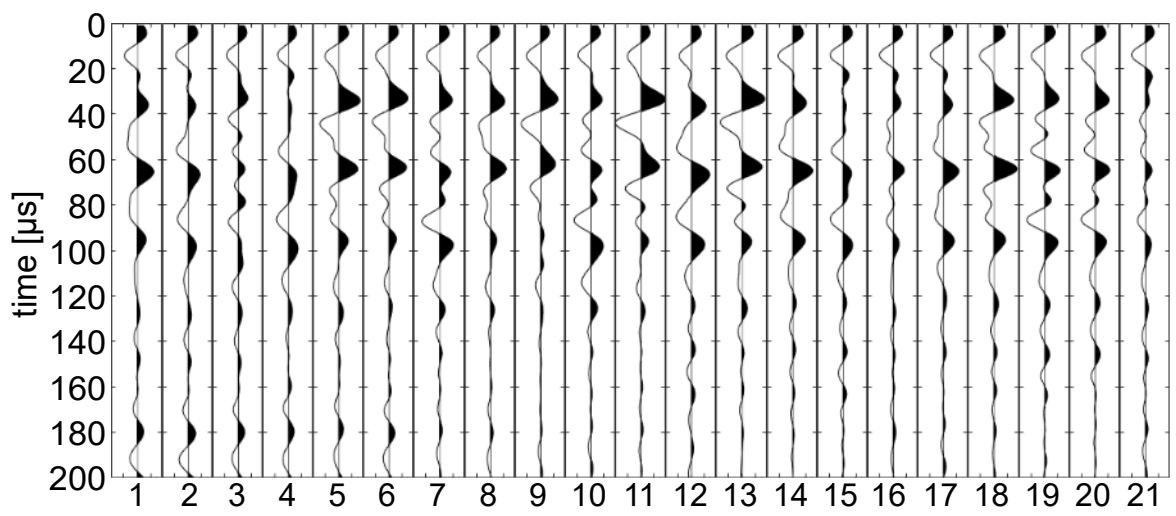

Profile 1
orientation
164/10
velocity
2687 m/s
1st layer
11.37mm±0.30mm
2nd layer:
25.21mm±0.67mm

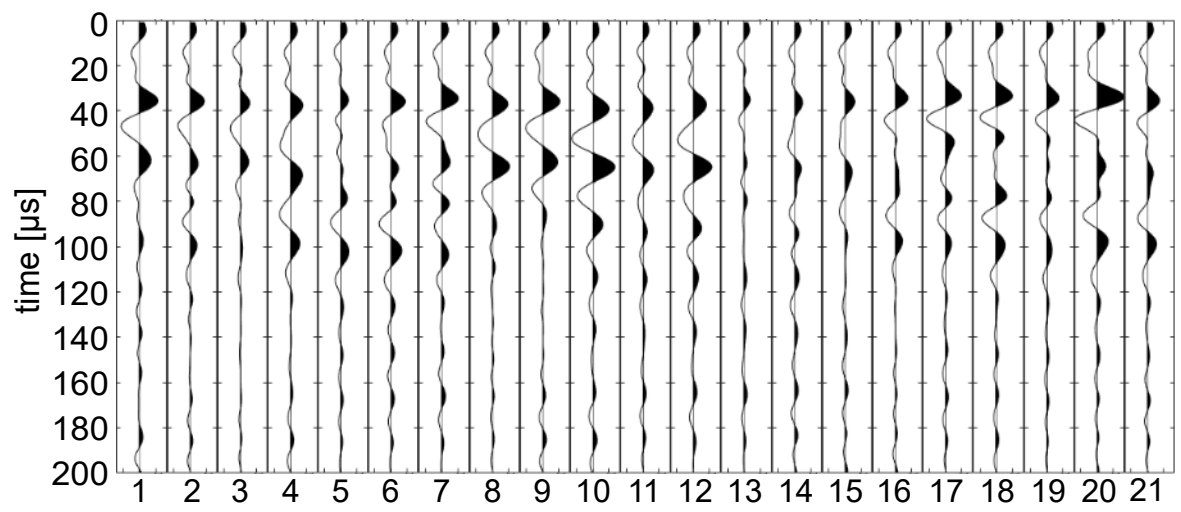

Profile 6
orientation
154/16
velocity
2744 m/s
1st layer
11.79mm±0.31mm
2nd layer:
26.12mm±0.70mm

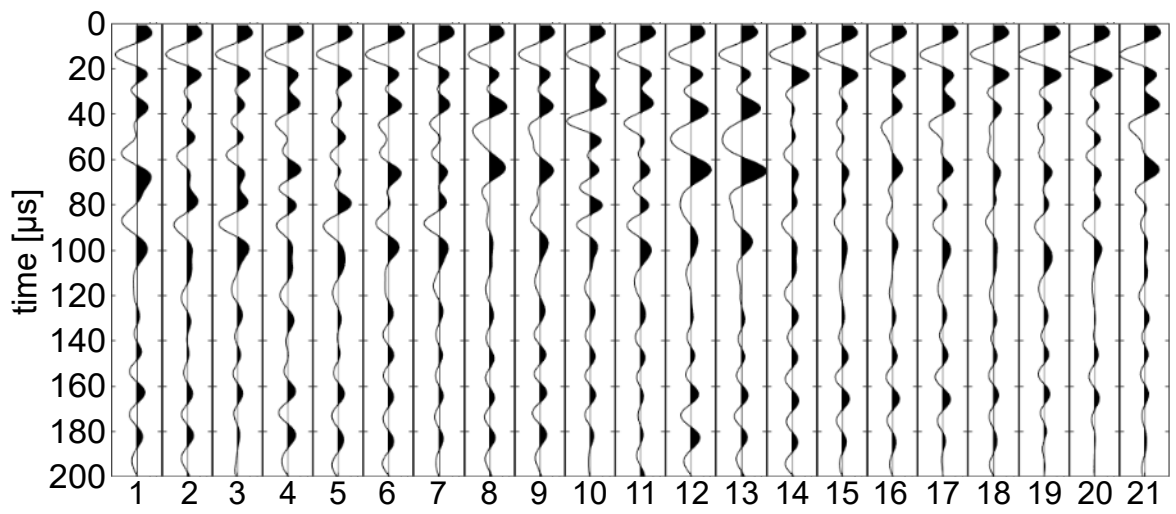

Profile 2
orientation
162/14
velocity
2687 m/s
1st layer
11.14mm±0.30mm
2nd layer:
24.89mm±0.66mm

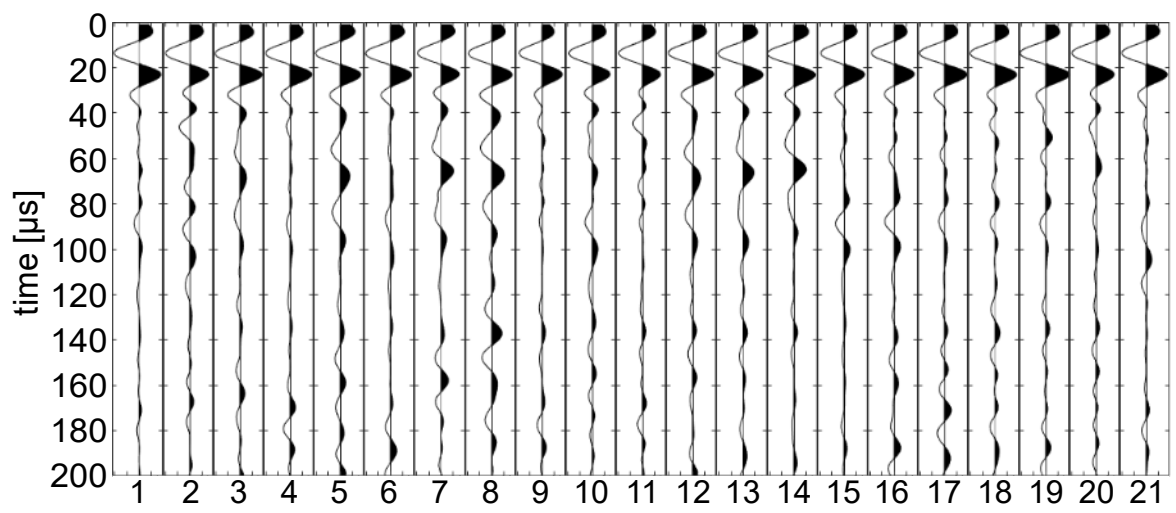

Profile 7
orientation
110/13
velocity
1869 m/s
1st layer
8.15mm±0.22mm
2nd layer:
17.93mm±0.48mm

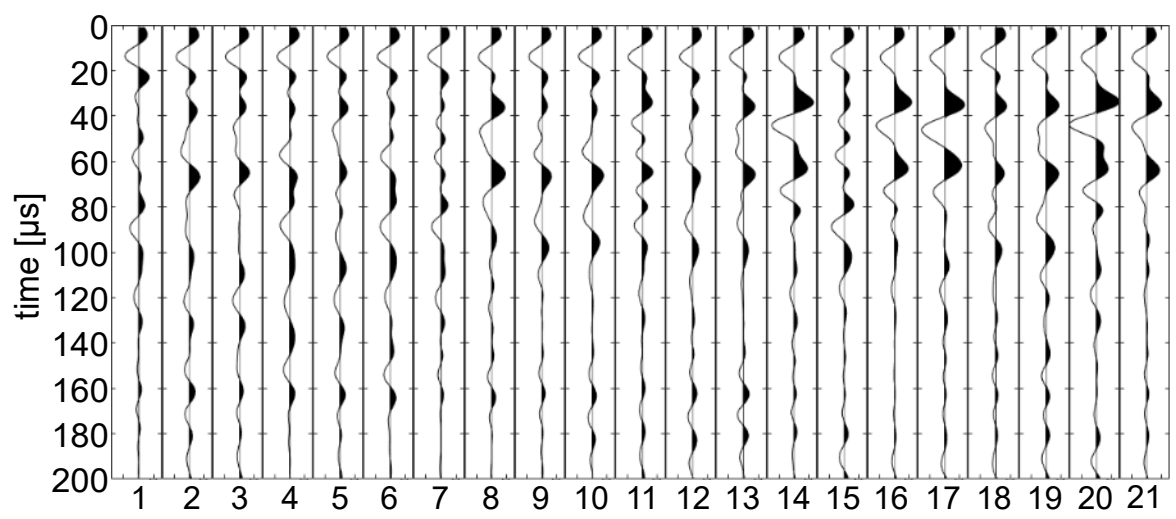

Profile 3
orientation
152/15
velocity
2687 m/s
1st layer
11.62mm±0.31mm
2nd layer:
25.49mm±0.68mm

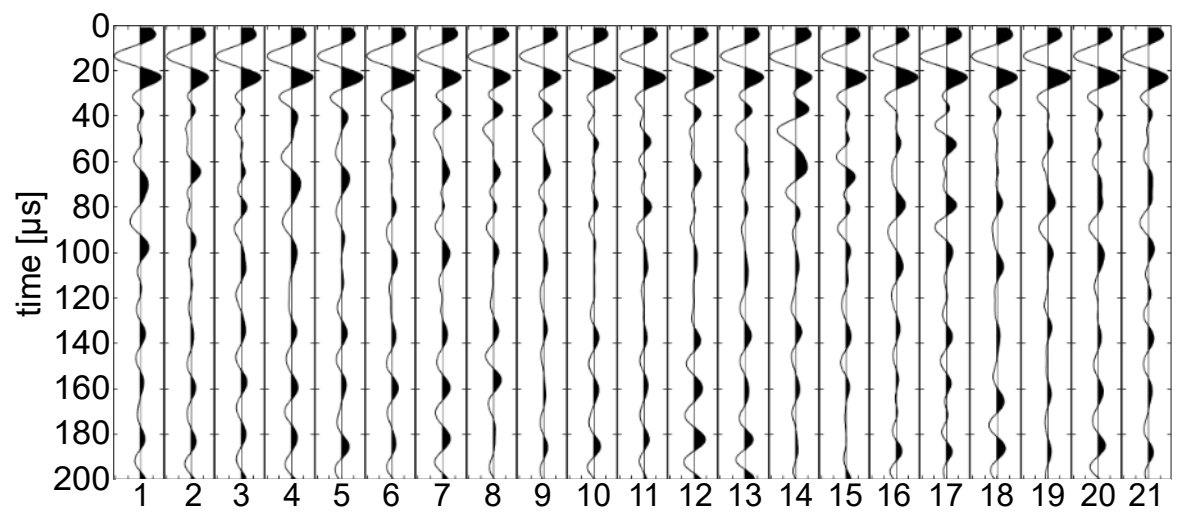

Profile 8
orientation
142/10
velocity
2769m/s
1st layer
12.04mm±0.32mm
2nd layer:
26.84mm±0.71mm

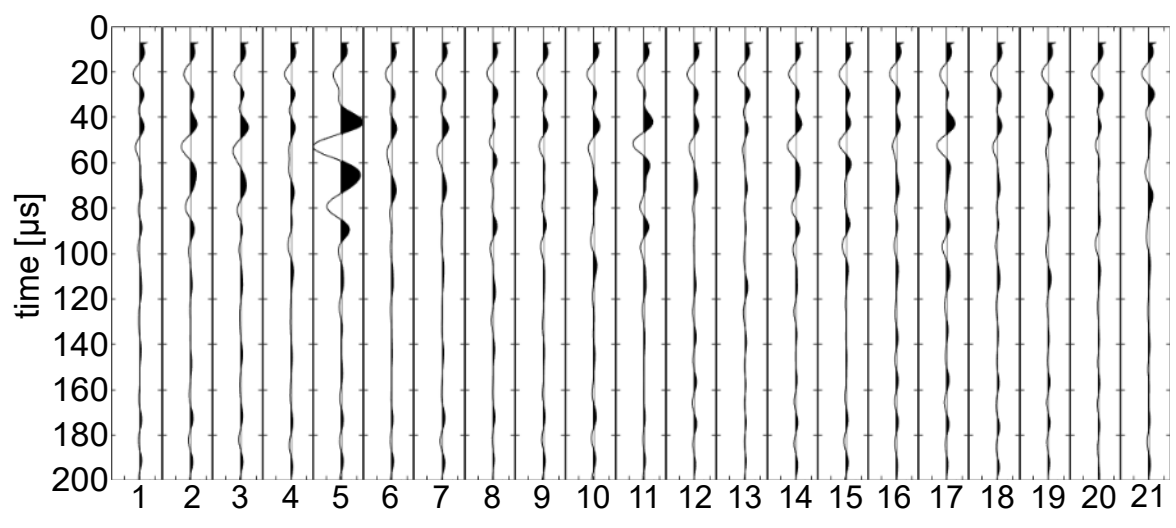

Profile 4
orientation
151/11
velocity
2744m/s
no layer

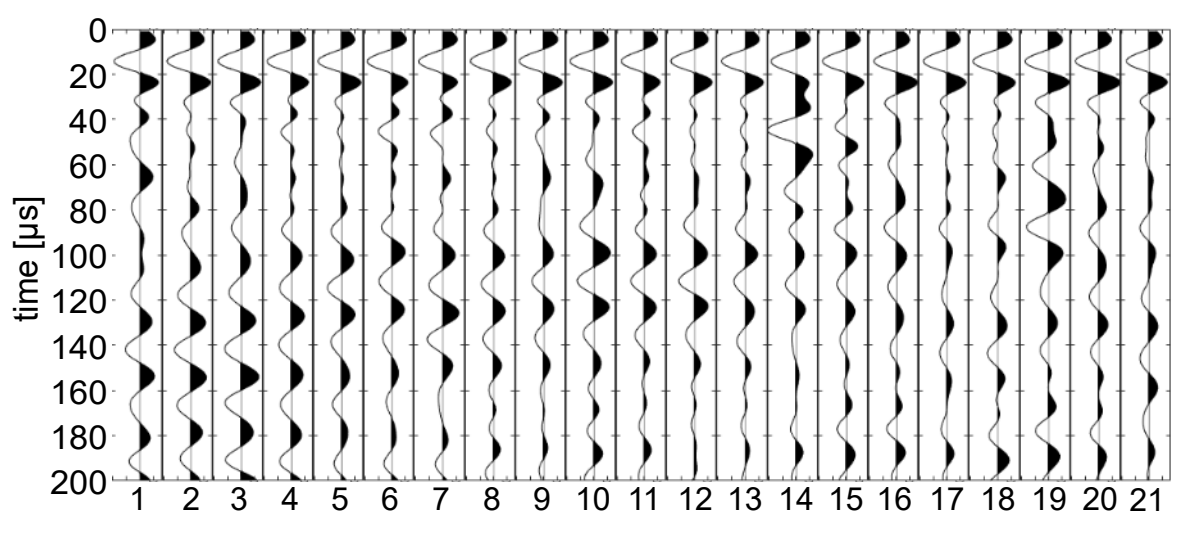

Profile 9
orientation
161/13
velocity
2769 m/s
1st layer
12.35mm±0.32mm
2nd layer:
26.71mm±0.69mm

Brastad 1:1

1st layer average
8.37mm±0.07mm
2nd layer average
18.67mm±0.17mm

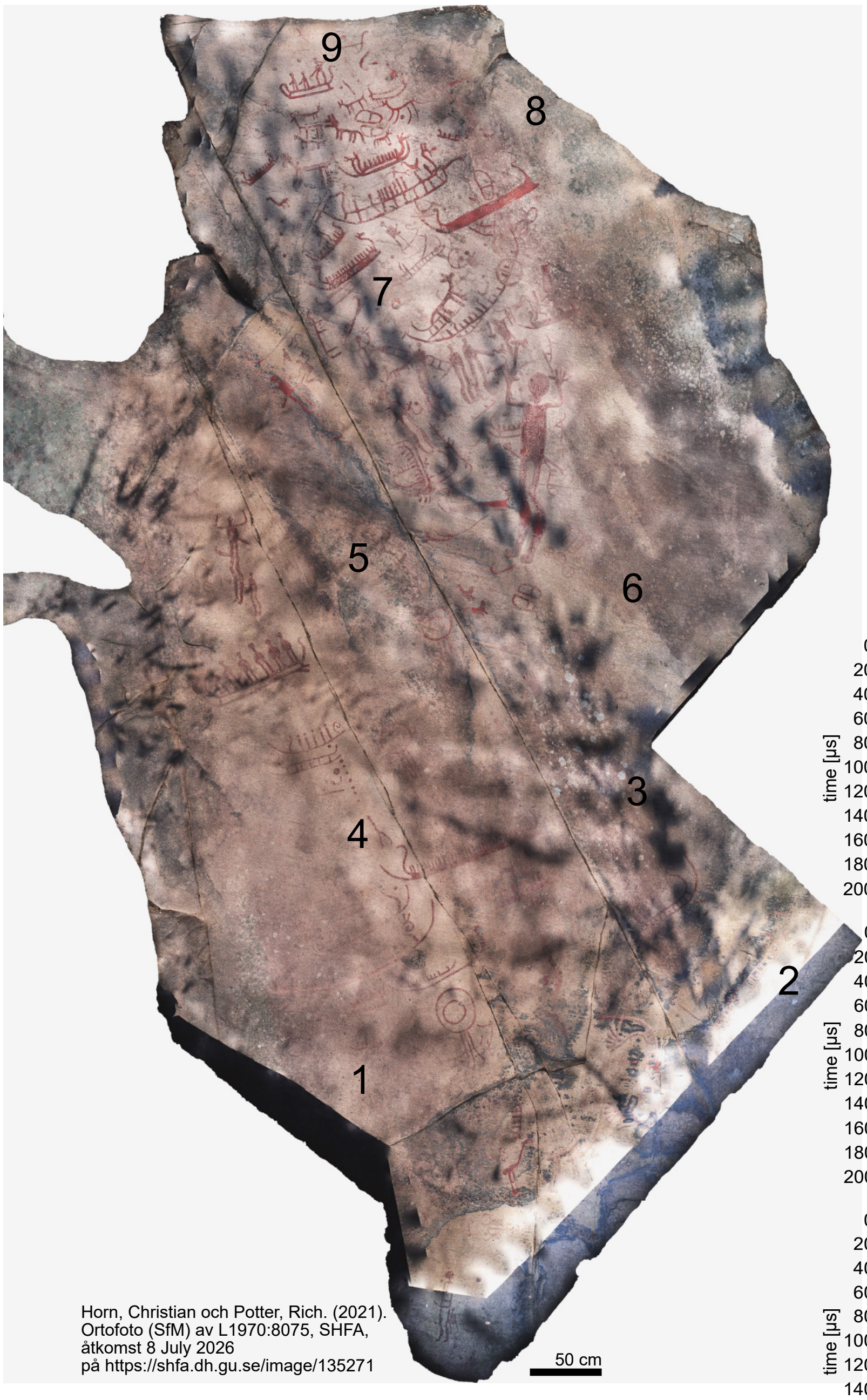

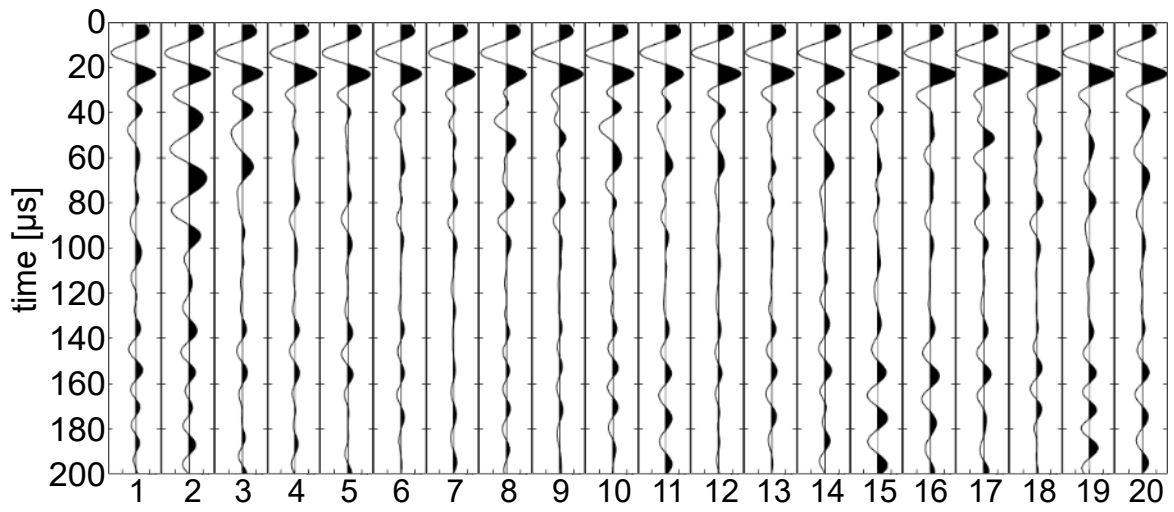

Profile 1
orientation
058/06
velocity
1995 m/s
1st layer
8.27mm±0.22mm
2nd layer:
18.42mm±0.50mm

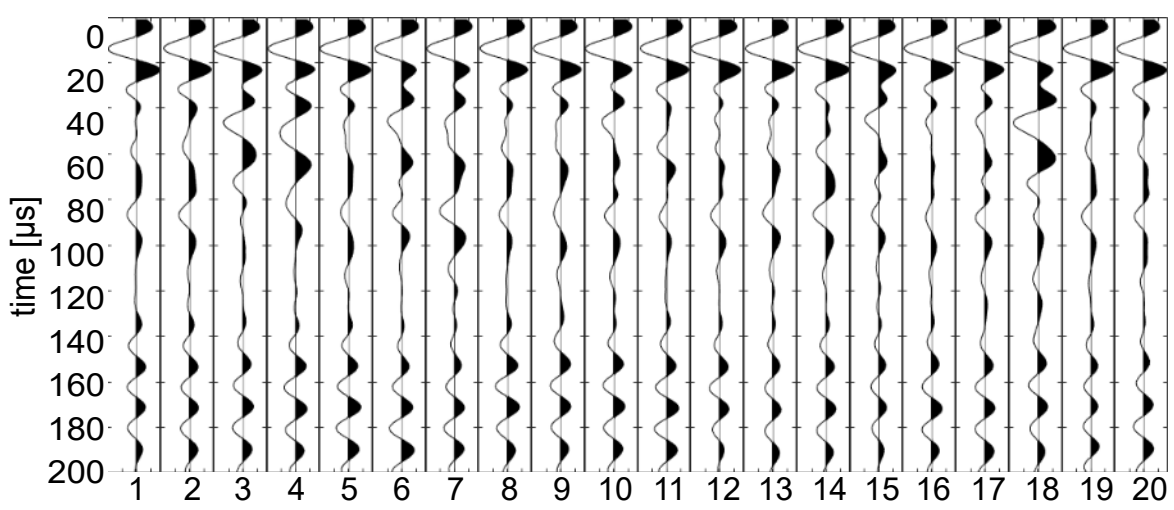

Profile 2
orientation
165/03
velocity
1995 m/s
1st layer
8.51mm±0.23mm
2nd layer:
18.90mm±0.50mm

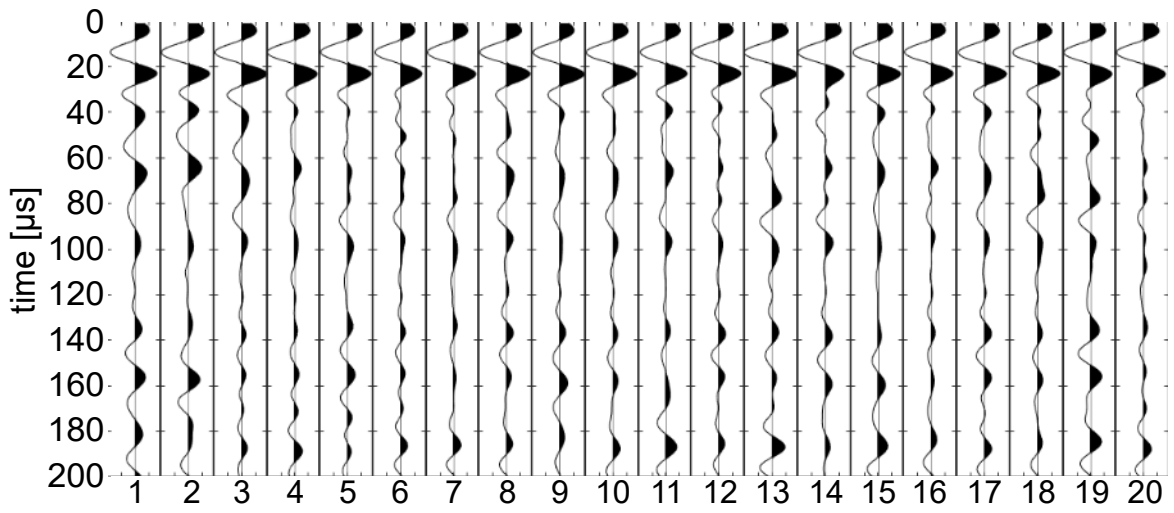

Profile 3
orientation
111/07
velocity
1995 m/s
1st layer
8.40mm±0.22mm
2nd layer:
18.70mm±0.50mm

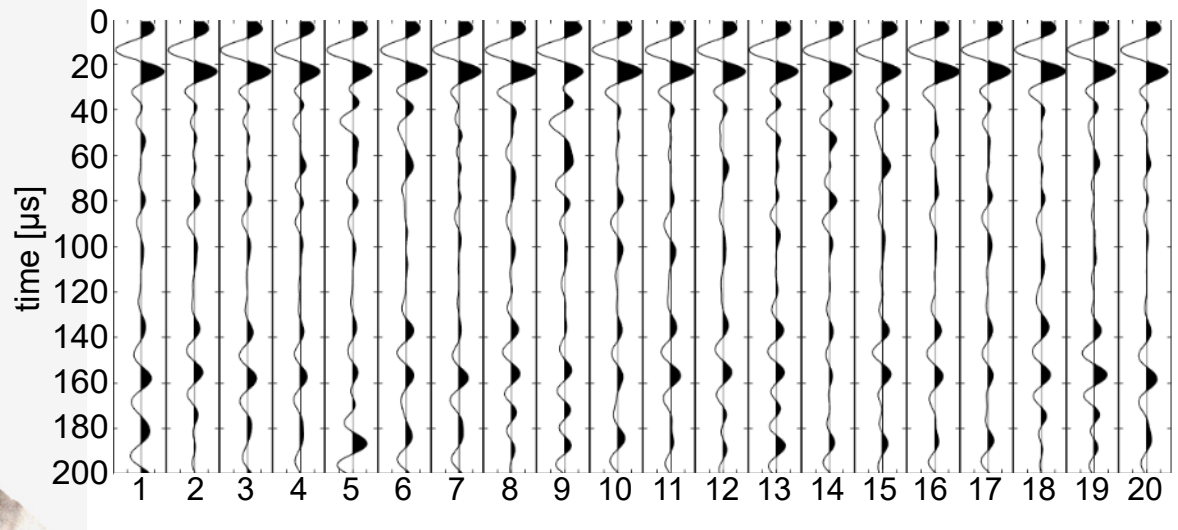

Profile 4
orientation
021/09
velocity
1995 m/s
1st layer
8.29mm±0.22mm
2nd layer:
18.57mm±0.50mm

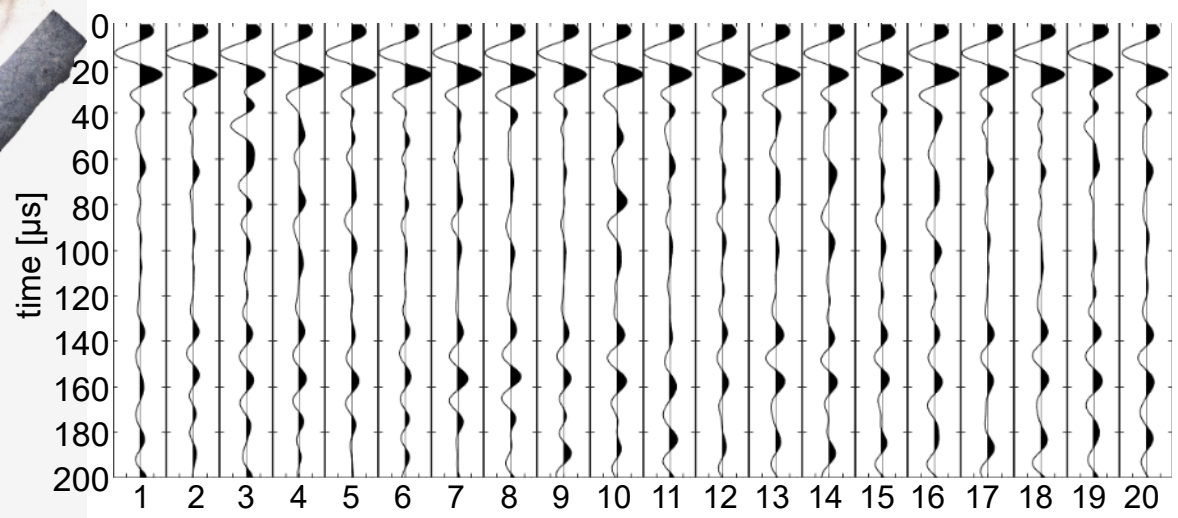

Profile 5
orientation
069/08
velocity
1995 m/s
1st layer
8.26mm±0.22mm
2nd layer:
18.49mm±0.50mm

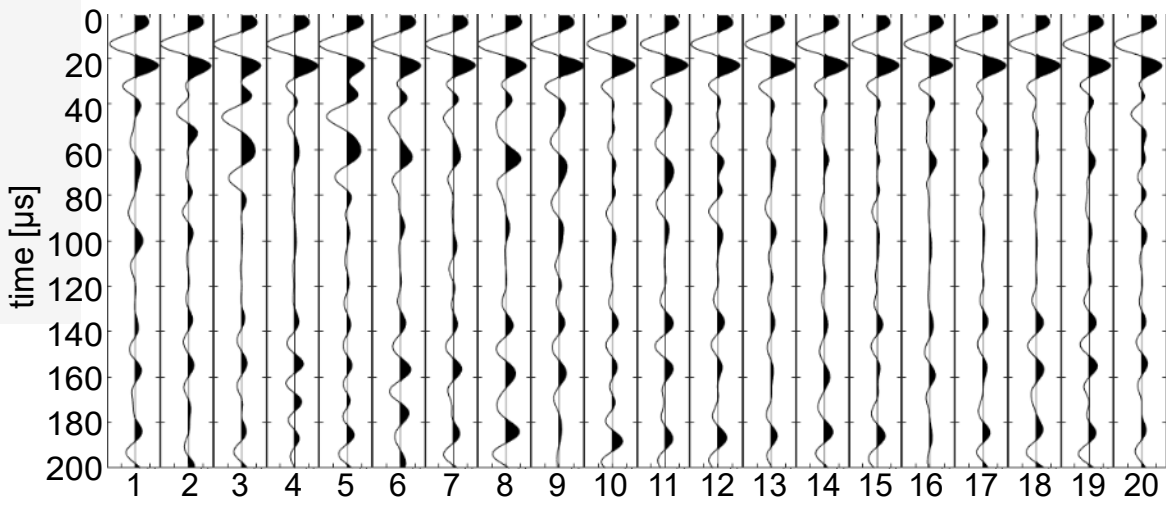

Profile 6
orientation
115/08
velocity
1995 m/s
1st layer
8.30mm±0.22mm
2nd layer:
18.63mm±0.50mm

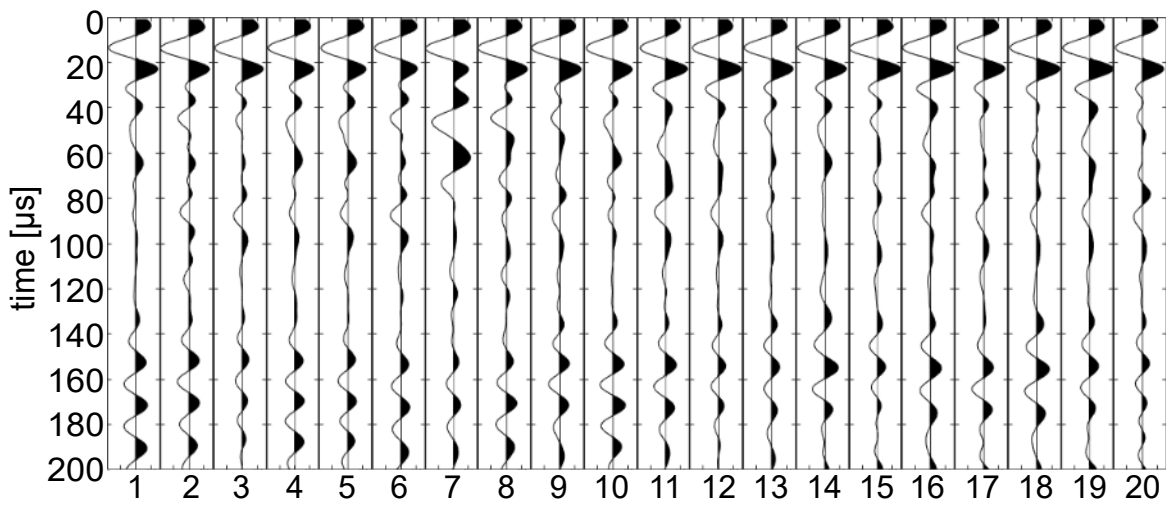

Profile 7
orientation
061/10
velocity
1995 m/s
1st layer
8.35mm±0.22mm
2nd layer:
18.65mm±0.50mm

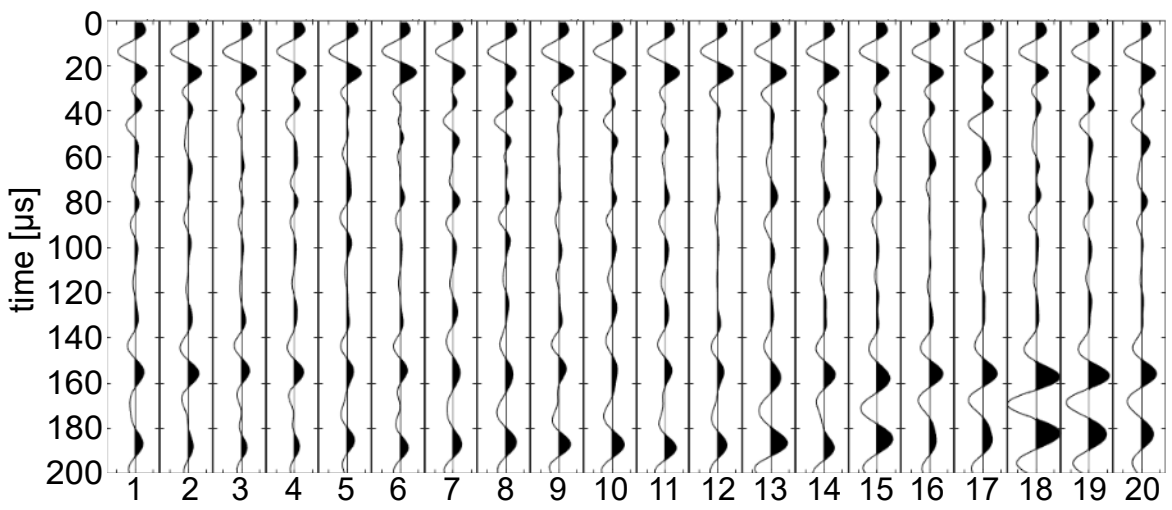

Profile 8
orientation
117/08
velocity
1995 m/s
1st layer
8.48mm±0.23mm
2nd layer:
18.79mm±0.50mm

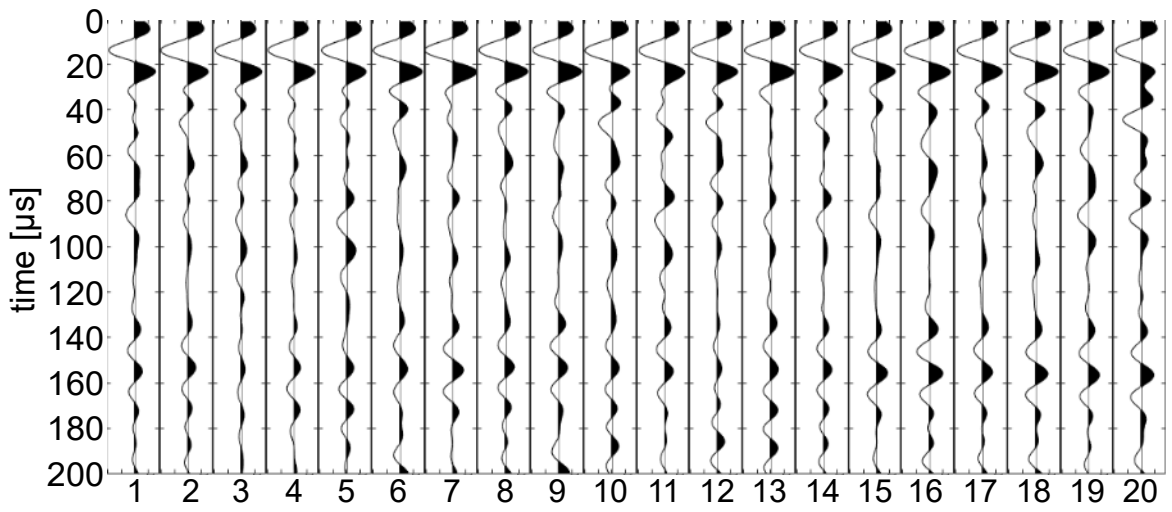

Profile 9
orientation
067/05
velocity
1995 m/s
1st layer
8.46mm±0.26mm
2nd layer:
18.77mm±0.50mm
